# Perceptions of a Secure Cloud-Based Solution for Data Sharing During Acute Stroke Care: Qualitative Interview Study

**DOI:** 10.2196/40061

**Published:** 2022-12-23

**Authors:** Marcela Tuler de Oliveira, Lúcio Henrik Amorim Reis, Henk Marquering, Aeilko H Zwinderman, Sílvia Delgado Olabarriaga

**Affiliations:** 1 Amsterdam University Medical Centers University of Amsterdam Amsterdam Netherlands

**Keywords:** qualitative interview study, electronic health records, cloud-based applications, acute stroke care, cross-organization data sharing, data privacy, encryption, data access control, mobile phone

## Abstract

**Background:**

Acute stroke care demands fast procedures performed through the collaboration of multiple professionals across multiple organizations. Cloud computing and the wide adoption of electronic medical records (EMRs) enable health care systems to improve data availability and facilitate sharing among professionals. However, designing a secure and privacy-preserving EMR cloud-based application is challenging because it must dynamically control the access to the patient’s EMR according to the needs for data during treatment.

**Objective:**

We developed a prototype of a secure EMR cloud-based application. The application explores the security features offered by the eHealth cloud-based framework created by the Advanced Secure Cloud Encrypted Platform for Internationally Orchestrated Solutions in Health Care Horizon 2020 project. This study aimed to collect impressions, challenges, and improvements for the prototype when applied to the use case of secure data sharing among acute care teams during emergency treatment in the Netherlands.

**Methods:**

We conducted 14 semistructured interviews with medical professionals with 4 prominent roles in acute care: emergency call centers, ambulance services, emergency hospitals, and general practitioner clinics. We used in-depth interviews to capture their perspectives about the application’s design and functions and its use in a simulated acute care event. We used thematic analysis of interview transcripts. Participants were recruited until the collected data reached thematic saturation.

**Results:**

The participants’ perceptions and feedback are presented as 5 themes identified from the interviews: current challenges (theme 1), quality of the shared EMR data (theme 2), integrity and auditability of the EMR data (theme 3), usefulness and functionality of the application (theme 4), and trust and acceptance of the technology (theme 5). The results reinforced the current challenges in patient data sharing during acute stroke care. Moreover, from the user point of view, we expressed the challenges of adopting the Advanced Secure Cloud Encrypted Platform for Internationally Orchestrated Solutions in Health Care Acute Stroke Care application in a real scenario and provided suggestions for improving the proposed technology’s acceptability.

**Conclusions:**

This study has endorsed a system that supports data sharing among acute care professionals with efficiency, but without compromising the security and privacy of the patient. This explorative study identified several significant barriers to and improvement opportunities for the future acceptance and adoption of the proposed system. Moreover, the study results highlight that the desired digital transformation should consider integrating the already existing systems instead of requesting migration to a new centralized system.

## Introduction

### Background

A stroke is a medical condition that occurs when the blood supply to a part of the brain is suddenly interrupted, classified as ischemic, or when a blood vessel in the brain bursts, spilling blood into the spaces surrounding the brain cells, classified as hemorrhagic [1]. Fast access to information is essential in acute stroke care. During an emergency, health care professionals from different organizations need to evaluate the patient’s condition, identify the type of stroke and severity, decide upon the treatment, transport the patient to the adequate care center, and perform the required intervention. Researchers have shown that the sooner the treatment is given, the better the outcomes for the patient are [2,3]. Moreover, patient transportation at the highest priority and hospital notification before patient arrival were associated with fast stroke care and better outcomes [4]. Finally, data availability through electronic medical records (EMRs) would improve decision-making and, ultimately, quality of care [5], leading to substantial reduction of unnecessary investigations and optimized communication among the acute stroke care teams involved in the treatment.

Emergency treatment of a patient usually requires cross-organizational collaboration: professionals at the emergency call centers, ambulance services, hospitals, and general practitioners’ clinics. In the Netherlands, these health care organizations are independent and have different policies and systems for patient data sharing. However, from the first call to the emergency call center, all the professionals involved need to exchange information while treating the patient. Currently, this information is exchanged orally or via phone, as there is no unified EMR that all professionals can share during treatment. Such conventional information-sharing methods consume time and effort, and they are prone to errors. Therefore, the need for a system that enables acute care professionals to share patient data throughout the treatment process is evident, despite the organization in which the professionals work. Such data also represent valuable sources of evidence for later medical research.

Cloud storage services provide an environment that matches the needs for remote and ubiquitous access to the patient’s EMR [6]. However, security and privacy challenges impede the widespread adoption of cloud services because they are susceptible to privacy and security threats [7]. Patients and health care organizations are afraid of losing control over the EMR when storing it on untrusted third-party clouds [8]. Finally, besides handling the privacy and security threats in cloud environments, cloud-based EMR applications must comply with the legal requirements regarding privacy and security imposed by the General Data Protection Regulation (GDPR) [9]. The GDPR attests that health care professionals and organizations are not obliged to systematically ask for patients’ consent before they can use the data contained in the EMR. However, the professionals are bound by all the principles described in Article 5 of the GDPR, which ensures that the exemption from consent is proportionate and limited to what is necessary for the patient’s treatment. Therefore, in the case of acute care, professionals are allowed to access the patient’s EMR only through their involvement in the treatment [10], requiring a solution that can dynamically grant and revoke access to the data.

A few solutions have been proposed to improve data availability and communication among professionals during acute care. Munich et al [11] presented a smartphone app to facilitate the tracking of the patient’s location during ambulance transfer between organizations. Nam et al [12] also proposed a smartphone app based on the Cincinnati Prehospital Stroke Scale to aid self-screening and hospital decisions. However, these apps do not provide access to the patient’s previous EMR.

Several studies have attempted to protect patient privacy in EMR cloud-based systems. Privacy-preserving approaches for eHealth clouds are classified as cryptographic and noncryptographic [10]. Various cryptographic approaches have been proposed to encrypt data in the cloud [13,14]. Seol et al [15] proposed a combination of approaches using attribute-based access control and encrypted files to share medical records stored in the cloud. However, these studies do not mention how to dynamically grant and revoke access to the encrypted data, which would be necessary to fully comply with GDPR.

Regarding dynamic access solutions, some systems offer *break-glass access*, which embodies the idea that, under certain conditions, a user can break the glass and explicitly override a denied access request [16]. Although some proposals use the break-glass approach to access encrypted EMR [17-20], access revocation after the emergency situation is still a problem. Thus, besides using encryption and access control to secure the data in the cloud, it is necessary to use modern techniques to adequately address all the requirements in acute care.

### The Proposed Acute Stroke Care Application

Advanced Secure Cloud Encrypted Platform for Internationally Orchestrated Solutions in Health Care (ASCLEPIOS) is a project funded by the Horizon 2020 program [21]. The project developed the ASCLEPIOS eHealth cloud-based framework, which deploys several modern cryptographic and access control mechanisms for protecting corporate and personal sensitive data. The framework enables and facilitates the development of cloud-based eHealth applications that can protect the patient’s privacy and prevent internal and external attacks. It combines dynamic index-based symmetric searchable encryption (DSSE) [22] and attribute-based encryption [23] to protect data in the cloud and to enable granting and revoking access to a user without interfering with the other users. These modern techniques allow dynamic management of encryption key access, therefore enabling more flexible access control that is important for acute care data sharing. Furthermore, the framework offers attribute-based access control based on flexible and configurable policies and attributes as an extra security layer to the encrypted data [24]. Only the users who hold the correct attributes can fulfill the policy and interact with the framework to access the data. Our organization participated in the ASCLEPIOS project and implemented a demonstrator exploring the framework for the acute stroke care case.

**Figure 1:**
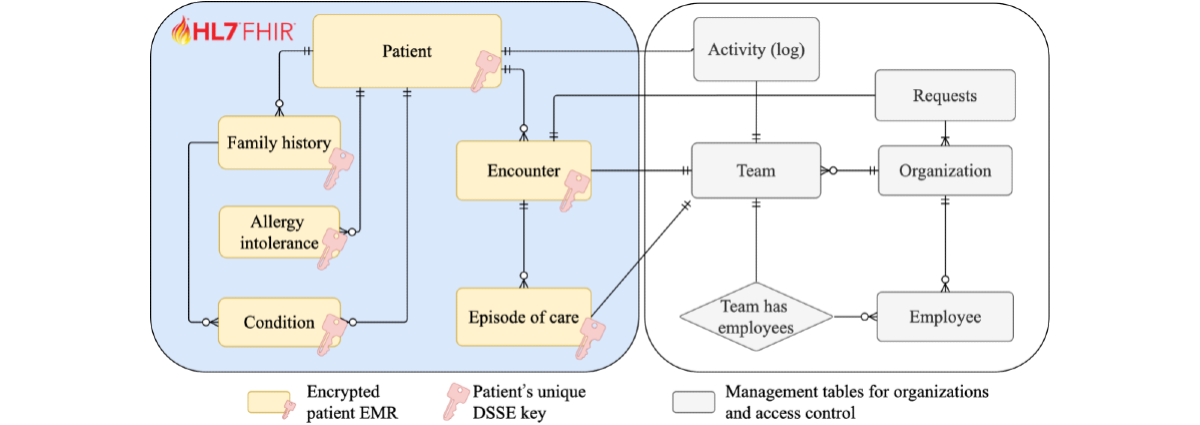
Electronic medical record (EMR) data model represented as entities relations of the Acute Stroke Care demonstrator, following the Health Level Seven Fast Healthcare Interoperability Resources (HL7 FHIR) standard. DSSE: dynamic index-based symmetric searchable encryption.

The ASCLEPIOS Acute Stroke Care demonstrator is a secure EMR cloud-based application that leverages the ASCLEPIOS framework to share data among the acute care teams in a cross-organizational paradigm. In particular, it ensures that a team only has access to the patient’s data under emergency conditions [25,26]. It relies on a unified EMR stored in the cloud in encrypted form to improve data accessibility during an emergency. Figure 1 shows the EMR data model, which follows the Fast Healthcare Interoperability Resources standard [27]. Figure 1 also shows the management entities and relations that the system uses to store necessary data, such as organizations, teams, and so on. Note that the EMR is encrypted with a unique key for each patient, and health care professionals can obtain access to the key and encrypted data only while treating that patient.

At the beginning of the project, we collected health care and data privacy requirements from the potential stakeholders: professionals from call centers, ambulance services, and hospitals. The requirement was first published by Chomutare et al [28] and Reis et al [29]. Table 1 summarizes the requirements for the Acute Stroke Care demonstrator.

**Table 1:** Summary of requirements of the Acute Stroke Care demonstrator (extracted from the study by Reis et al [29]).

Requirement	Description
Availability	EMR^a^ should always be available for access by legitimate users.
Confidentiality	Only authorized users should access the EMR.
Integrity	The accuracy and consistency of the EMR should be assured.
Nonrepudiation	The professional cannot deny what they have done.
Auditability	For every action, it must be possible to know who did it and what, when, where, why, and how the action occurred.

^a^EMR: electronic medical record.

We implemented a web-based application to address the requirements listed in Table 1, with functionality to strengthen users’ trust and comply with the GDPR. The EMR data are encrypted using a combination of DSSE to protect the data and attribute-based encryption to protect and manage the DSSE keys. The implemented attribute-based access control policies grant and revoke health care professionals’ access according to their participation in the patient’s acute stroke care timeline and present the EMR through the professionals’ user interfaces.

**Figure 2:**
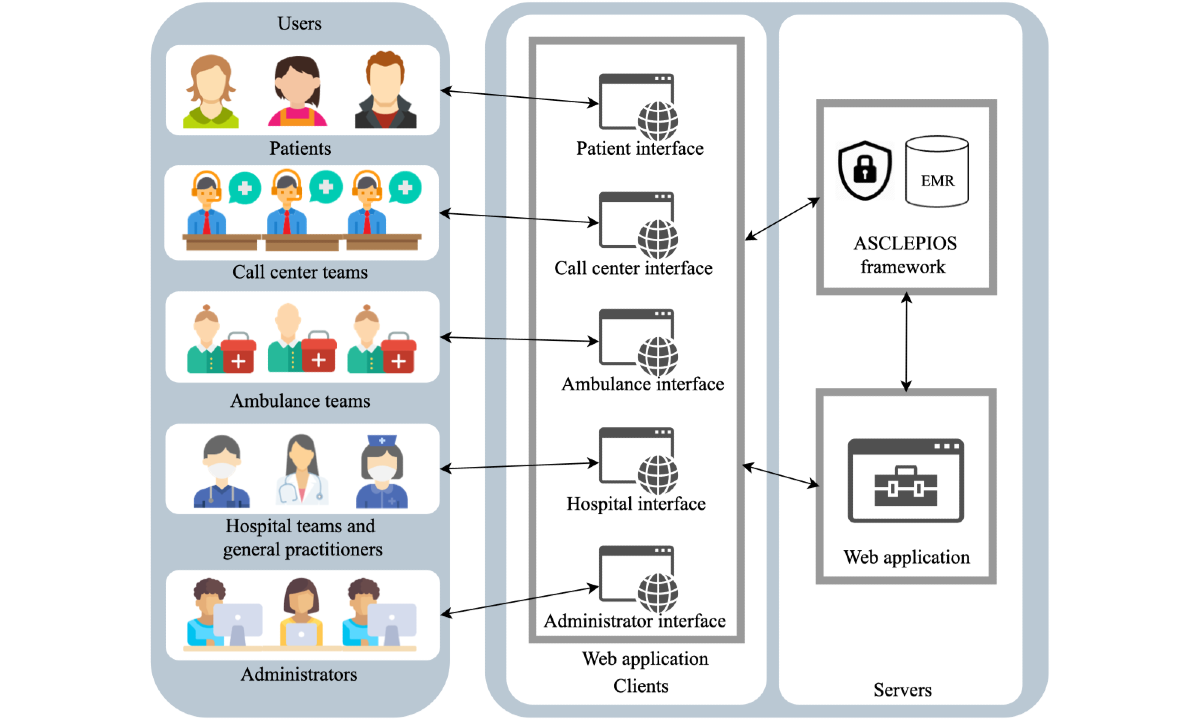
Diagram of the Acute Stroke Care demonstrator architecture with the Advanced Secure Cloud Encrypted Platform for Internationally Orchestrated Solutions in Health Care (ASCLEPIOS) framework and the stakeholders involved. EMR: electronic medical record.

Figure 2 shows a diagram of the architecture of the Acute Stroke Care demonstrator with the ASCLEPIOS framework and the stakeholders involved (patients and health care professionals). Patients and health care professionals have their own interface, through which they can interact with the system in different ways.

**Figure 3:**
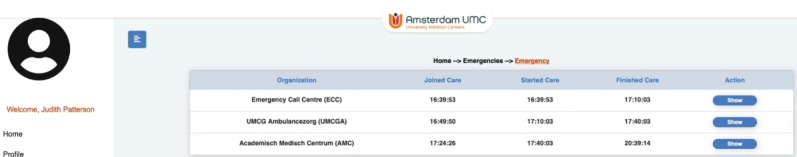
Example of the patient interface showing the organizations that treated them in some past emergency.

We implemented a specific user interface where the patients can add their medical conditions, allergies, medications, and family history; read data added by health care professionals; and visualize data access logs. Figure 3 shows an example of the patient interface with the list of organizations that treated them in a past emergency. For each organization, there are time stamps from when the organization joined, started, and completed acute care. 

**Figure 4:**
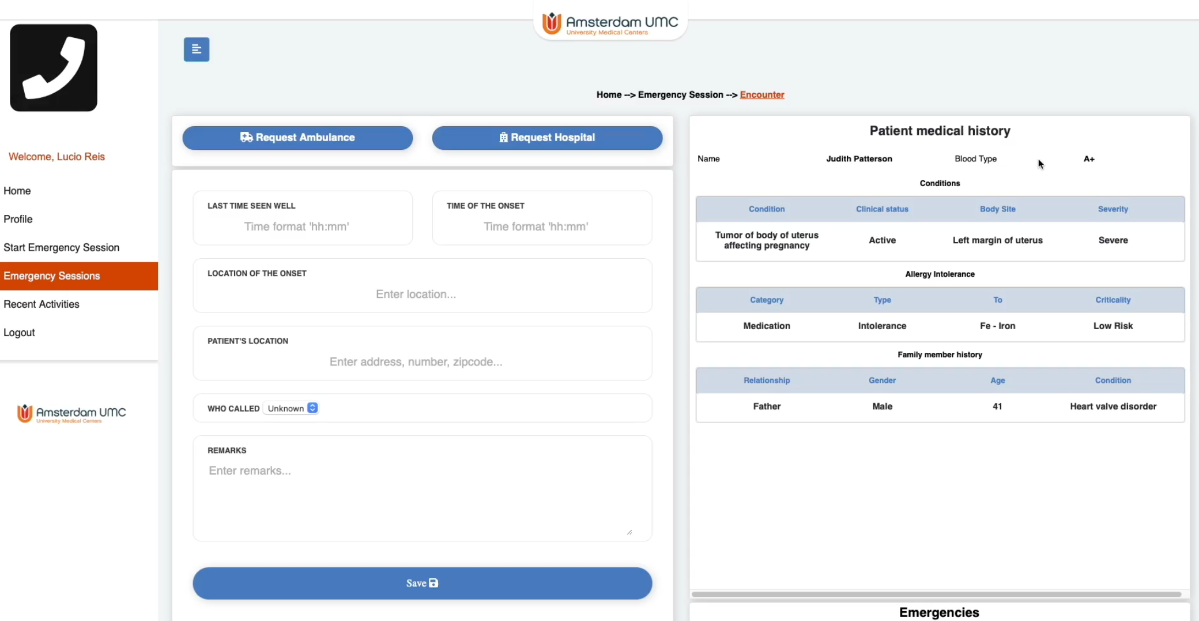
Example of call center interface treating a patient during an emergency.

For each role in each organization, there is an interface through which, during an emergency, the professionals can access the patient’s EMR and request other teams to join the emergency. Figure 4 shows an example of the call center interface used to treat a patient. The call center can input relevant information and request another team (eg, ambulance team), and on the right side, the EMR of the patient is presented. The interfaces for the ambulance and hospitals are similar to that shown in Figure 4.

More information about the application can be obtained from our previous study [25] and videos in Multimedia Appendices 1 and 2.

**Figure 5:**
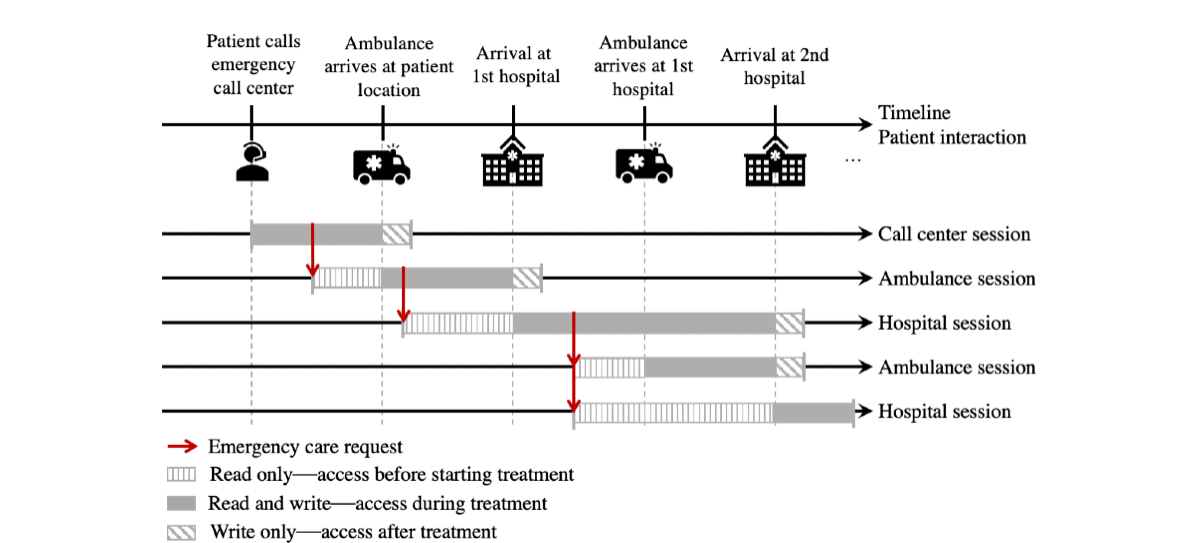
Example of an acute stroke care timeline involving multiple health care organizations.

Figure 5 illustrates the information flow considered in the application during an emergency session, starting when the patient has a stroke until treatment completion at a hospital. An emergency session is the interval of time when all access to the patient’s EMR occurs during acute care. The teams involved in the treatment become part of the emergency session for a period and leave the session when their task is completed. In this case, the patient contacts the emergency call center for help. From this moment, the call center professional searches for the patient’s identification in the EMR system and starts an emergency session for this patient. Next, the call center professional requests an ambulance team to participate in this emergency session. After the ambulance arrives at the patient’s location, the ambulance team performs triage and decides the hospital to which the patient must be taken for treatment. Once they know which hospital to go to, the hospital team also becomes involved in the emergency session of the patient. After arrival at the hospital, the hospital team confirms or invalidates that the patient has experienced an ischemic stroke and performs adequate treatment. The patient is finally discharged and returns home. The same procedure occurs if the call center cannot identify the patient in the system. In such a case, a temporary identification is used to store and share the patient’s data during the emergency, and later, the data are merged into the patient’s EMR.

Figure 5 highlights that the health care professionals of each organization are involved only for a limited period, and access to the patient EMR must be provided only when necessary, complying with the GDPR. In an acute stroke care scenario, an involved health care team requests the participation of another team in the treatment; for example, the call center requests an ambulance to pick up the patient. Given the urgency, for adequate preparation, it would be necessary for the new team to have access to read the patient’s EMR even before meeting the patient; for example, the requested ambulance team can read the patient’s history during displacement. Moreover, the teams should have extra time to add data that could not be input during the treatment. Finally, access to the EMR must be revoked for any team that no longer participates actively in the patient’s treatment; for example, access by the call center team is revoked after the ambulance team picks up the patient.

### Significance

It is essential to gain user input early in technology development to improve applications according to users’ needs [30]. In this study, we presented the stakeholders with a web application designed to facilitate patient data sharing among acute care professionals using a secure cloud solution. We also explained how this application would be used during a simulated scenario of acute stroke care. This presentation served to disseminate a new vision for secure data exchange during a medical emergency, where the data are encrypted and decrypted locally in the user’s device before being sent to the cloud. Moreover, access to patients’ data is granted and revoked dynamically to the professionals according to their participation in the treatment. Furthermore, this study aimed to raise awareness and attract stakeholders’ interest in this type of service. Finally, the stakeholders’ impressions and feedback further validated the ASCLEPIOS Acute Stroke Care application concept, thus providing valuable input for further technology development.

### Objective

The goal of the interviews was 2-fold. First, the goal was to show the application’s use to the main stakeholders: professionals from emergency call centers, ambulance services, and emergency hospitals and general practitioners. Second, we aimed to collect their impressions about how the application would fit into their daily acute care workflow.

### Research Questions

With this study, we aimed to answer the following research questions (RQs):

- RQ1—What are the current challenges in patient data sharing during acute stroke care?
- RQ2—What are the participants’ impressions about the proposed ASCLEPIOS Acute Stroke Care application?
- RQ3—What would be the challenges and suggestions for the adoption of the ASCLEPIOS Acute Stroke Care application in a real-life scenario?

## Methods

### Overview

We conducted an in-depth interview–based study with the main stakeholders in acute stroke care. We started recruiting participants and requesting their consent to record the interviews. The interviews were divided into 3 parts. First, we asked about the participants’ familiarity with cybersecurity tools for data sharing in questionnaire part A. Second, we presented the ASCLEPIOS framework concepts and a simulation of the use of the ASCLEPIOS Acute Stroke Care application during acute stroke care and by the patient. Third, we asked about the participants’ impressions regarding the use of the application in questionnaire part B. We tailored the in-depth interview according to the answers to the questionnaire, and the discussion evolved based on emerging findings. We conducted a qualitative thematic analysis of the data collected through the questionnaires and transcriptions of the interviews.

### Recruitment

Participants were recruited from 4 groups, namely, representatives of emergency call centers (group 1 [G1]), ambulance services (group 2 [G2]), and emergency hospitals (group 3 [G3]) and general practitioners (group 4 [G4]). We started recruiting potential participants via email based on a contact person from the Amsterdam University Medical Center. Each message introduced the project and requested for an interview. Interviews were scheduled with those who responded and provided informed consent to participate. After an interview, we always asked if the participants could indicate other potential participants from the 4 groups. We sent a total of 19 invitations. A follow-up email was sent to nonresponders after 1 week. When we did not get any response, we stopped any further contact with nonresponders, assuming that they had no interest in participating.

The recruitment process and interview occurred in 3 phases from September 2021 to August 2022: the first phase with 43% (6/14) of the participants, the second phase with 36% (5/14) of the participants, and the third phase with 21% (3/14) of the participants. We stopped recruitment when we reached thematic saturation and had similar representation of the 4 main stakeholders and potential users of the application. Our study’s theoretical saturation refers to the point in data collection when no additional themes or insights are identified and data begin to repeat so that further data collection is redundant, signifying that an adequate sample size is reached [31]. During the second phase, we reached thematic saturation. In the third phase, we validated the saturation once the participants did not bring any new themes or suggestions in addition to those already put forward by participants in the previous phases.

### Data Collection

In the study, 2 coauthors interviewed each participant individually. Of the 14 participants, 9 (64%) participants were interviewed in person and 5 (36%) were interviewed via the web. In general, the interviews lasted approximately 45 to 60 minutes. We interviewed participants from various acute care organizations in the Netherlands. During the interviews, we collected data of 2 types: the answers to the structured questionnaire (parts A and B) implemented using Google Forms (Google LLC) [32] and the audio recordings of the interviews conducted via a cell phone. All the demographic data collected are stored in a private file. Table 2 summarizes the demographic information about the interviewees.

**Table 2:** Demographics of the participants (N=14).

Variables		Count, n (%)
**Sex**		
	Male	9 (64)
	Female	5 (36)
	Intersex	0 (0)
**Role in acute care**		
	Emergency call center professional	3 (21)
	Ambulance nurse	4 (29)
	Emergency and neurologist physicians in hospital	4 (29)
	General practitioner	3 (21)
**Experience in acute care (years)**		
	0-4	2 (14)
	5-9	4 (29)
	10-14	1 (7)
	15-19	3 (21)
	20-25	1 (7)
	≥25	3 (21)
**Region in the Netherlands**		
	North Holland	9 (64)
	Utrecht	3 (21)
	South Holland	2 (14)

### Data Management

After the interviews, we transferred the recordings via a secure private network to the *otter* service to automate the transcription process [33]. The transcriptions were treated according to the 6 steps proposed by Azevedo et al [34]. Interview transcripts, notes, and answers to the questionnaires were pseudonymized using the same identifiers and divided into 4 groups. For example, “Participant 1 from G1” is a professional from an emergency call center. The audio recordings were stored in an encrypted digital audio recorder maintained in a local machine. Only the pseudonymized transcripts were shared with other coauthors. The audio recordings will be retained for 1 year after the end of the ASCLEPIOS project (June 2023), and the transcripts and answers to the questionnaires will be retained for 5 years after the end of the project.

### Data Analysis

Data were analyzed following the 4 steps from the principles of qualitative study and systematic text condensation [35]. This procedure consists of the following steps. First, we read the transcripts and the answers from the questionnaires to obtain an overall impression and identify preliminary themes as responses to the RQs of this study. The preliminary themes were directly related to the questionnaires. Second, we defined the coding that represented the themes and subthemes. Then, we read all the transcripts and answers once again and assigned themes and subthemes to the transcripts, with the support of *MAXQDA* software (VERBI GmbH) [36]. Third, we condensed the transcripts and answers into themes and subthemes. Finally, we synthesized the descriptions of the participants’ impressions and their feedback as quotations.

### Ethical Considerations

All participants were asked to provide written consent based on oral and written information about the study, and only those who provided their consent were included (14/19, 74%). The study did not collect or otherwise handle patient-related or health-related data. All the data collected using the questionnaires through Google Forms were pseudonymized and correlated to the transcripts through the time stamps. Moreover, only the authors (MTO and LHAR) had the permission to access the data in Google Forms. The ASCLEPIOS project’s ethics advisory committee and data protection officer assessed the study design and informed consent forms. They concluded that a more rigorous ethical review was unnecessary because the study did not collect any sensitive or personal data.

## Results

### Overview

A total of 14 participants were interviewed. They classified their roles as professionals from call centers (3/14, 21%), ambulance services (4/14, 29%), hospitals (4/14, 29%), and general practitioners’ clinics (3/14, 21%). We represent the 4 groups to show the diversity of the participants according to their roles in acute care. In general, the interviewees were very interested in understanding the vision proposed by the application and were excited to provide feedback.

We identified 5 themes in the data analysis, namely, current challenges (theme 1), quality of the shared EMR data (theme 2), integrity and auditability of the EMR data (theme 3), usefulness and functionality of the application (theme 4), and trust and acceptance of the technology (theme 5). In the analyses phase, we did not observe any significant correlation between the groups and answers, and there was no theme that was mentioned only by a specific group. Therefore, the results are not presented per group, and we only use the groups in the citation because it provides more context to participants’ quotations.

An overview of the identified themes and subthemes is presented in Textbox 1. Table 3 presents the relationship among the identified subthemes, the questions from the questionnaires (parts A and B), and this study’s RQs. The results presented in the following subsections use the questionnaire part and the number of the question; for example, A1 is the answer to questionnaire part A, question 1.

**Overview of themes and subthemes.**

**Theme 1**

- Current challenges
- Subthemes
  - 1.1—The current systems lack standardization and structure of data
  - 1.2—Noninteroperability of systems hampers the exchange of data
  - 1.3—Achieve professionals’ awareness about security and privacy of the patients’ data

**Theme 2**

- Quality of data
- Subthemes
  - 2.1—Reliability of the data provided by the patient
  - 2.2—Reliability of the data provided by other teams

**Theme 3**

- Integrity and accountability
- Subthemes
  - 3.1—Prevention of data loss
  - 3.2—Accountability of the data added and edited during the treatment
  - 3.3—Duration of the extra time to add and edit data after the end of treatment
  - 3.4—How to handle unknown patients during acute care

**Theme 4**

- Usefulness and functionality
- Subthemes
  - 4.1—Integration of the application with other (exiting) systems as data sources
  - 4.2—Granularity of access control to parts of the electronic medical record
  - 4.3—Information about the patient’s condition after the treatment, for learning purposes

**Theme 5**

- Trust and acceptance of the technology
- Subthemes
  - 5.1—Professionals’ training to use the system
  - 5.2—Extend the system to include all types of stakeholders of an electronic medical record system
  - 5.3—Merge current systems instead of proposing a new one
  - 5.4—Increase patient trust and awareness

**Table 3:** Questions from questionnaires (part A and part B), how they are related to the research questions of this study, and the identified subthemes.

Questionnaire part and questions		Research questions		Subthemes
**A**				
	1. Do you use any EMR^a^ system to share patient data?	1	1.1 and 1.2	
	2. Is the EMR system cloud-based?	1	1.1, 1.2, and 1.3	
	3. Is the patient data encrypted in the EMR system?	1	1.1 and 1.3	
	4. Would you be willing to share encrypted patient data in a cloud-based solution across multiple healthcare organisations?	1	1.1 and 1.3	
	5. How important is it to keep the patients’ data confidential and only available to the healthcare professionals involved in their treatment?	1	1.3	
	6. How much would a patient data leakage affect the patient’s life?	1	1.3	
**B**				
	1. How would information such as medical conditions, allergies/intolerances, and family history, as informed by the patient in the demo, be useful in case of emergency?	2	2.1	
	2. How much would the availability of patient data before the treatment improve the decision-making during treatment?	2	2.2	
	3. Do you believe that a digital system, such as the demo, could prevent data loss?	2	3.1 and 3.2	
	4. The demo considers accountable the professional, the team, and the organisation who added new data to the patient record during treatment. Who do you think should be accountable?	2	3.2	
	5. Do you think that healthcare professionals should be able to add or edit the patient’s data after the treatment ends?	2	3.1, 3.2, and 3.3	
	6. Do you think a system like this demo could be useful in a real situation?	2	3.4	
	7. What would be needed to improve the usefulness of a system like this demo?	3	4.1, 4.2, and 4.3	
	8. Would you trust using a system like this demonstrator in your daily tasks?	3	5.1	
	9. What would be needed to increase your trust in a system like this demo?	3	5.1	
	10. How likely would your organisation be to accept adopting a system like this demo in a real situation?	3	5.2 and 5.3	
	11. What would be needed to improve your organisation’s acceptability of a system like this demo?	3	5.2 and 5.3	
	12. Do you think a system like this demo could make patients feel safer about providing their data to your organisation?	3	5.4	

^a^EMR: electronic medical record.

### Current Challenges for Patient Data Sharing During Acute Stroke Care

The first theme emerged when the participants answered questionnaire part A. All participants (14/14, 100%) told us about how they share patient data during acute care and their difficulties. Of the 14 participants, 13 (93%) said that they use EMR systems to share patient data and feel comfortable with them (A1). Overall, one-third (4/14, 29%) of the participants use cloud solutions, one-third (5/14, 36%) do not use the cloud, and the remaining one-third (5/14, 36%) do not know how the system stores the data (A2). Most participants (12/14, 86%) use different systems in different organizations, and these systems usually do not communicate directly with each other (subtheme 1.1). In the Netherlands, the call center and ambulance professionals can share data about the emergency. However, these professionals do not have access to previous medical records; they have access to data only about the ongoing acute care event. The hospitals usually do not communicate directly with the ambulance systems, and the data are generally duplicated when shared. Moreover, in North Holland, the ambulance team can print the information collected during patient transportation and give the paper to the hospital team on arrival. A participant expressed this as follows:

...Now we are still working in such an old fashion with paper. Even after the team types the information inside the ambulance, I will receive a paper printed out or a PDF document when I receive the patient. Then I need to manually extract what I think is relevant information and insert it into another system with 10-15 words, and this is the medical report in the patient file.

Participant from G3

The lack of interoperability was also mentioned as a big challenge because, even if they have access to other systems, they usually cannot merge the patient data into a single EMR (subtheme 1.2). The general practitioners have to merge the records manually when following up on the patient’s treatment:

As a GP [general practitioner], when my patient calls and I suspect that there is a stroke, I will request an ambulance, and I will receive a notification when the patient arrives at the “hospital x” and receives the treatment. But I can’t see anything more. So I need to ask them for the treatment records, and I receive a PDF file again, and I need to insert the information again into the GP system. This is really annoying!

Participant from G4

The participants told us about their awareness of security and privacy responsibilities regarding the patients’ data (subtheme 1.3). Of the 14 participants, 10 (71%) do not know if their EMR system stores the patient data in encrypted form (A3). Nevertheless, all participants (14/14, 100%) were willing to share encrypted patient data in a cloud-based solution across multiple health care organizations (A4). In addition, they all agreed that it is important to keep patient data confidential and make them available only to the health care professionals involved in their treatment (A5). Of the 14 participants, 13 (93%) believed that patient data leakage would affect the patient’s life (A6). Some of them also criticized the current data management approaches, which usually offer break-glass buttons that bypass the conventional access control mechanism of the system to any professional who has access to the system:

When I need to access some data that I usually don’t have access to, a “break-glass” pop-up appears, and if I click yes, I have access to the data.

Participant from G3

### Participants’ Impressions About the Proposed Application

The second theme emerged when the participants answered questionnaire part B, regarding their impressions about the application after seeing it in use.

The application enables the patient to input some information into the system, such as medical conditions, allergies, intolerances, and family history. Therefore, we asked how such information could be useful in an emergency case. Of the 14 participants, 13 (93%) believed that it would be very much useful (B1). However, all the participants (14/14, 100%) commented on the doubts about the sufficient quality and reliability of the information provided by the patient for acute care decision-making (subtheme 2.1):

Usually, when patients add medical information to their files, that is not the type of information that a doctor is looking for. For example, if patients add that they have a tumour, they cannot say the location of the tumour nor describe it as the doctor will do. Thus, the information is not that useful, but it is better than nothing.

Participant from G3

As a doctor, I don’t think that the data the patient inputs to the system is 100% reliable. I would trust it more if another doctor had added the information.

Participant from G4

Although all participants (14/14, 100%) agreed that the availability of data before the treatment starts could improve decision-making (B2), some types of data are double-checked and input into the system again when the patient is delivered to another health care team, for example, when the ambulance delivers a patient at the hospital (subtheme 2.2):

Having access to what the teams [call center and ambulance] added about the patient can save a lot of effort and make the treatment faster. However, suppose the patient comes from another hospital and has already done some imaging. Nowadays, the next hospital team usually remakes the images exams even if they have access to the previous exam.

Participant from G4

Well, it’s great that the emergency nurses write down what’s going on. As a doctor at the end of the line, I would already know the blood pressure of the patient or something. But the truth is that it is very likely that we are going to check them again.

Participant from G3

The third theme emerged when we asked the participants’ perspectives about how much a system such as our application could prevent data loss (subtheme 3.1). In theme 1, the participants mentioned that the lack of interoperability makes them rewrite essential data, and much information is lost in this process. During the interview, all participants (14/14, 100) mentioned that using a centralized system would prevent data loss (B3):

...Prevent data loss? The central system on itself? Yes, absolutely.

Participant from G1

...We can prevent this [data loss] when we all use one platform, and it is secure like a cloud [referring to our application].

Participant from G2

Moreover, we asked the participants who should be accountable for the data added to the EMR of the patient when a team treats the patient (subtheme 3.2). All of them (14/14, 100%) agreed that the person who added the data is accountable, but 71% (10/14) of the participants thought that the whole team should also be responsible and traceable for what happens to the patient, as proposed in the demonstrator (B4):

The accountability of the data is what makes the doctor remake the image exams. They do not trust that the image was made correctly in another hospital, so they need to double-check before deciding or giving a diagnostic and writing it down.

Participant from G4

Every professional involved in the treatment should be accountable and traceable.

Participant from G1

All the professionals who participate in the treatment should be accountable, but the professional who wrote the data must be responsible for it.

Participant from G2

Furthermore, we asked how long access to patient data should still be provided after the treatment is over, for example, to input data that could not be added earlier owing to the urgency of the treatment or other responsibilities (subtheme 3.3). All participants (14/14, 100%) agreed that the data should be added as soon as possible to be useful to other teams involved in the acute care, but they also agreed that, sometimes, the extra time is fundamental to complete and edit all the forms. Of the 14 participants, 9 (64%) believed that a few hours are enough as extra time, whereas 5 (36%) considered a few days (B5):

At the end of our shift, my colleagues and I always go back to the reports. We write any information that we haven’t added because of the hurry. So, I believe 24 hours is a good extra time, more than that is too much.

Participant from G3

This is a difficult question. Because when I look into my practice, sometimes it happens that we arrive at the hospital, we deliver the patient. And then they call us again, and we have cardiac arrests around the corner, then we don’t have time...Of course, it is not a standard procedure, but this happens quite often. So, I think if the team needs extra time, they should click the button saying that they need to keep the session open until the end of their shift and close it as soon as possible.

Participant from G2

Because we make mistakes when we type the information, we should be able to fix them when we have time. But I think that access after the treatment is over must be logged as editing data.

Participant from G2

We asked if the participants thought that a system such as our demonstrator would be useful in their daily tasks. All of them (14/14, 100%) responded that it would be useful, and 79% (11/14) said that it would be very useful (B6):

...The cloud solution itself will be very useful. All the [user] interfaces are not, but for the cloud solution, definitely yes.

Participant from G1

Of the 14 participants, 5 (36%) highlighted that, sometimes, the patient cannot be rapidly identified to obtain the existing medical records in the system. They were very interested in the application’s function that enables the system to store the data generated in the treatment using crypto scheme and later merge these data with the patient’s EMR (subtheme 3.4):

...Sometimes when there is a tourist, for example, it takes some time to find their ID or passport or whatever. So then, it would be handy to be able to merge that [patient data] afterwards.

Participant from G2

### Challenges and Suggestions for the Adoption of the Application

The fourth theme emerged when we asked what would be needed to improve the usefulness of the system. The participants made various suggestions to enhance the usefulness and functionality of the application (B7).

The participants suggested that the application should include other types of care, such as regular physician appointments, which would require the admission of more types of users in the application and extend the access control model to cover their requests. At least, the system should be able to exchange data with other (existing) systems (subtheme 4.1):

I think one of the things that I missed is that you can push information to your base to the local EMR system.

Participant from G4

The participants provided feedback regarding the granularity of access control to parts of the EMR (subtheme 4.1). Overall, 36% (5/14) of the participants suggested that the system should support splitting the patient’s EMR into 2 parts—one part of data that is shared with the patient and another part of the data that is shared among the health care professionals. This 36% (5/14) of the participants believed that the patient should not read all the annotations that the health care professionals create. They mentioned that physicians write information about triage, which needs further investigation to remember what was done before the diagnosis. According to them, such information should only be shared among the health care professionals involved with the treatment. They affirmed that this type of information could create misunderstanding and unnecessary stress for the patients. In contrast, all participants (14/14, 100%) agreed that patients should be able to read about the diagnosis and procedures performed during treatment:

Nowadays, patients have access to part of the data. I add to the EMR only the diagnostics and measurements. I also add some notes to the patient. However, I have another place to add my comments as a doctor. For example, if a suspect that the patient has cancer, I do not add this in his report directly. First, I ask for exams, but I need to keep this note to remember the patient’s case with more details.

Participant from G4

Another 21% (3/14) of the participants said that patients should be able to read all the data about their treatment and they should be informed as much as possible:

So now [in the demonstrator], the patients can see anything I type. So now, I think I will sometimes be very careful. On the other hand, if you type it down, you can also say to the patient. If you can’t say it to the patient, so maybe you shouldn’t write it down. If you say, if you write down the patient is maybe faking it, you should also tell the patient that you think he is faking it. So yeah, I think anything I typed down is also something I would tell the patient. Yeah. I don’t know if other doctors think otherwise. This is kind of a regulation thing. I believe. The patient has some will on this.

Participant from G3

Of the 14 participants, 4 (29%) suggested that the application should include more data sharing opportunities for learning purposes (subtheme 4.2). These participants said that they are interested in performance measurement, such as aggregated metrics about the organizations. Others were interested to know more about what happens after they leave the patient under the care of other teams, mainly to learn whether their decision was correct:

...Can you get aggregated metrics, for example? Because this is what we need to report, some hospitals and departments, like the entry of emergency departments. Or, for instance, for ambulances, to report how fast they were for every patient with stroke because this is like a quality metric that we have to show to improve the quality of the service.

Participant from G3

You’re not a taxi when you transfer the patient in an ambulance. I believe that the professionals involved in the treatment should see what happens with the patient even after their task is done because it is part of the learning process.

Participant from G2

In the fifth and last theme, we analyzed the trust and acceptability of the application among the participants and the challenges regarding its adoption in a real scenario. All participants (14/14, 100%) said they would “much” and “very much” trust using the application in their daily tasks (B8). Overall, 64% (7/11) of the participants highlighted the need to train health care professionals to use a digital system such as the demonstrator (subtheme 5.1). Once the professionals understand how the system works and its security scheme, they will trust and be motivated to use it (B9):

...The point is that human errors happen pretty often because the professionals are not able to interact with the [current] system. When things go wrong in the hospital [system], that affects the patients negatively. Thus, the professionals must be trained to use the system correctly.

Participant from G3

Of the 14 participants, 13 (93%) believed that their organization would adopt a system such as this application (B10). To improve the acceptance by health care organizations (B11), 57% (8/14) of the participants suggested that our application should include more types of users beyond the acute care teams and offer opportunities for data sharing among all of them (subtheme 5.2):

This system should be able to comprise other types of access, so we extend the security measures that you created for acute care to include the conventional and all the other types.

Participant from G4

The feedback obtained from 71% (10/14) of the participants was to think about integrating the existing EMR systems with the ASCLEPIOS framework (subtheme 5.3). All of them (14/14, 100%) seemed to value the application, but they also reinforced that the acceptance of a new centralized national EMR system would be far-fetched. Therefore, the recommendation was to consider using the framework as an interoperability layer between the existing systems:

The organisation is very sceptical about new systems, so this can be a barrier to the organisation’s acceptance. But if we prove that the system works properly and if it could be interoperable with the existing system, it would help the process.

Participant from G1

...If you want all the acute care workers to work in the same system, that won’t be easy. But if they would work in their systems and connect all those systems with web-based applications or anything else we did with this cloud solution that will be there, then there is a fair chance that it can work.

Participant from G2

When they [acute care professionals] have to write down everything into [multiple] systems, it’s too much. So they don’t do it. I think the very important thing is that this system is the only one they need to work with.

Participant from G4

Finally, all participants (14/14, 100%) answered that patients would feel safe about sharing their data (B12). However, 64% (9/14) of the participants said that most patients are not aware of the privacy risks related to EMR leakage. Therefore, 14% (2/14) of the participants suggested that health care organizations should be more transparent about the patient data processing and create awareness about privacy risks (subtheme 5.4):

I think most of the patients are not thinking at this level. Most of the patients are not thinking about their privacy risks or if their data is available in case of an emergency. They usually think about it after something happens.

Participant from G1

It depends on the medical records of the patient. If he [patient] is applying for a job, but he had a heart problem once, maybe he will be concerned about what the company would say if they illegally already know.

Participant from G2

## Discussion

### Principal Findings

The main objective of this study was to collect the current challenges for patient data sharing during acute stroke care (RQ1), the participants’ impressions of the proposed ASCLEPIOS Acute Stroke Care application (RQ2), and the challenges and suggestions for adapting the ASCLEPIOS Acute Stroke Care application in a real-life scenario (RQ3). Although our study was designed in the context of a specific European Union project, the challenges of developing an EMR system that supports acute care and the collected feedback about cloud-based systems are applicable in a broad context.

From the results for RQ1, this study reinforced that the most relevant challenges for patient data sharing are the lack of interoperability and connectivity between systems from different organizations. For RQ2, this study obtained relevant feedback from every interviewee regarding the time interval for data availability, accountability, prevention of data loss, and handling of unknown patients during acute care. For RQ3, this study identified several important barriers to and improvement opportunities for the future acceptance and adoption of the proposed system.

Furthermore, this study aimed to validate the security concepts of a cloud-based medical data sharing application for acute stroke care that exploits the ASCLEPIOS framework. During the interviews with health care professionals, it became evident that they experience—daily—the lack of a properly connected and secure information infrastructure for patient data exchange across organizations. The application was well received and considered to be relevant by all participants (14/14, 100%). However, as a large number of noninteroperating systems are used in practice, replacing them with a new system—such as the developed application—did not seem realistic. An alternative path to be explored involves developing an interoperation layer for cloud-based security and trusted data exchange that could bridge legacy systems with the newly developed technology.

Another interesting finding is that the participants were excited to provide feedback when we said that we would demonstrate the usefulness of our project in a simulation to support acute stroke care. We simulated the workflow, emphasizing that the professionals from each team could access the patient EMR only from the moment when they were invited to participate in the treatment until their tasks were completed. Thus, they could see the added value that the proposed solution could bring to facilitate data sharing among all the professionals involved. Furthermore, the received feedback validates the access control model implemented in the application.

Finally, we highlight 3 suggestions that the participants provided to increase the usefulness of the system and regarding what we could achieve using the ASCLEPIOS framework. The first suggestion was to expand the system to support all types of access to EMRs. The second suggestion was to create more granularity of access control for different types of data contained in the EMR, which would require separating the data that are sharable with the patient from those that are shared only among the health care professionals. The third suggestion was about consulting aggregated metrics from all the EMRs stored for learning purposes. All these suggestions provide valuable feedback that will be explored in future studies.

### Limitations

A limitation of the study is that demonstrating the use of application interfaces can be a double-edged sword. In addition to seeing how the system would work and understanding the solution behind the screen better, the participants may also be distracted by the interfaces presented during the simulation. We anticipated this effect, and thus, we stimulated participants to provide feedback beyond the user interface. Nevertheless, we still received suggestions about interface content and design modifications, which were not relevant to this study’s RQs, but they could be useful in a future application design.

Moreover, we acknowledge that collecting the perspectives of hospital administrators and technical staff is essential for accepting the new health care system. Therefore, in the future, we will design a study to collect their perceptions and feedback from management and technical perspectives.

Another limitation was related to the COVID-19 pandemic. To perform in-depth interviews, we preferred to have in-person meetings and let the participants interact with the application. However, acute care professionals are very busy, and even more so because of the pandemic; thus, it was even harder than anticipated to involve the professionals in person. Moreover, there were multiple lockdowns during the study; therefore, we had to use web-based meetings to prevent the cancellation of the already confirmed interviews. Regarding these web-based interviews, we realized that, unfortunately, the communication and interaction were limited because they could not directly visualize the application being used. Besides this limitation, the 36% (5/14) of the participants provided valuable feedback during the web-based meetings.

### Comparison With Previous Studies

Researchers have successfully adopted similar sociotechnical qualitative interviews to collect stakeholders’ perceptions and validate the concept of innovative technological solutions for health care. 

Murry et al [37] interviewed senior managers and medical staff to explore and understand their experiences of implementing eHealth initiatives and their assessment of factors that promote the integration of eHealth initiatives. In total, 23 interviews were conducted, and they showed substantial differences in the implementation of eHealth initiatives [37]. It differed from our study because their focus was not on health professionals’ perspectives. Instead, the authors interviewed the implementers, who are the staff responsible for implementing digital eHealth systems, which, according to the authors, is an under-studied group. Moreover, the implementers showed rich understanding of the barriers to and facilitators of successfully implementing such initiatives. 

Georgiou et al [38] also conducted a qualitative interview study to assess the impact of introducing new health technical initiatives for medical imaging processing. They used a mixed methods study design comprising semistructured interviews with medical imaging department staff and retrospectively extracted emergency data. In the study by Georgiou et al [38], the results show that the accessibility of images and patient-related information improve the efficiency of the medical imaging department. In our study, the professionals also agreed about the potential improvement in efficiency by having the data available from other teams. Moreover, similar to the study by Georgiou et al [38], in subtheme 2.2, professionals raised concerns about the quality of the data, especially the reliability of the image data provided by other teams in acute care. 

Similar to our results, the studies by Murray et al [37] and Georgiou et al [38] affirm that for the successful implementation of an eHealth system, it should be a good fit between the new technology and existing skill sets or efforts made to teach the requisite skills to users. Similarly, in our study, professionals recommended integrating the new application with other (existing) systems (subtheme 4.1) and merging current systems instead of proposing a new one (subtheme 5.3). 

Azode et al [39] conducted a qualitative interview study to investigate the opportunities for and challenges of using data from wearable sensor devices in health care. In total, 16 health care, technology, business, innovation, and social sciences experts were interviewed in a qualitative, theoretically informed study. The authors concluded that current applications cannot fulfill their potential if they do not yield benefits for clinical users and integrate effectively with the existing eHealth systems. In our study, health care professionals were interested in expanding our system’s application to include all types of EMR data, which could also include data from wearables. 

Hasselgren et al [40] interviewed medical students and analyzed their perceptions of a blockchain-based decentralized work for maintaining professional history and credentials portfolio. The study used a qualitative approach applied with data collection through 9 semistructured interviews. The results showed that health care professionals are interested in a decentralized system in which they can control their credentials and reputation.

Brandt et al [41] interviewed patients who are overweight to identify important drivers of long-term personal lifestyle changes from a patient perspective when using a collaborative eHealth tool. Interviews were conducted 5 years after the initial intervention and showed that all the patients still used other internet apps to benefit their health despite not having access to the eHealth tool used during the intervention.

Although the objectives of the applications used by Hasselgren et al [40] and Brandt et al [41] differ from EMR data sharing, our application has a common goal—to increase the trust on eHealth systems among patients. For this aim, our application presents to the patient a consolidated logs dashboard about how the patient data were processed by health care professionals. In the study by Hasselgren et al [40] and our study, health care professionals are not sure how aware the patients are about the digital systems and how effective these functionalities of health care transparency are, but in the study by Brandt et al [41], patients show trust and value in the use of the proposed eHealth app. This reinforces subtheme 5.4, which recommends increasing patient trust on and awareness about digital health systems and applications.

Woodward et al [42] explored the personal experiences of health care professionals using eHealth innovations for data sharing in selected postconflict situations. This study used a cross-sectional qualitative design, with 12 telephone interviews. The authors concluded that all interviewees held positive perceptions that the eHealth system can help them to access information and communicate with other health workers. However, understanding of the scope of eHealth was generally limited and often based on innovations that health workers have been introduced to by their international partners. In our study, health care professionals also raised concerns about the need for training to use eHealth applications. In the study by Woodward et al [42] and our study, the results show the importance of training so that professionals can accept and benefit from the eHealth innovation system.

Inspired by previous studies [37-42], we used similar methods and acknowledged the importance of gaining stakeholders’ input for eHealth technology development, for further improvement and acceptability of new technologies.

In our previous study [29], we collected and analyzed the perspectives of medical staff regarding health care and data privacy requirements for the eHealth cloud, using a qualitative interview. At that time, we collected requirements that would guide the design of the demonstrator. Moreover, we investigated the participants’ understanding of cloud services and how they envision using the ASCLEPIOS solution in their daily tasks. At that point, we did not have the Acute Stroke Care application ready to present to the clinicians.

In this study, besides validating the requirements discussed in the previous publication [29], showing the participants a working application allowed them to go deep into the matter and ask questions related to the actual usefulness and acceptance of the ASCLEPIOS solution for cross-organization acute stroke care data sharing.

### Conclusions

This study validated the need for a cross-organization data sharing solution that offers the security and privacy required when patient data are processed. The participants emphasized that our cloud-based application would solve the data sharing problems, such as duplication of data, lack of information, and standardization. However, it would not be realistic to propose that all the organizations involved in acute care migrate to a unique cloud-based application. Future studies should investigate opportunities to update the system according to these inputs and further explore the ASCLEPIOS framework as a secure and interoperable layer for patient data sharing. The concept validation and feedback presented in this study incite the desire for a digital transformation in health care systems.

## Appendix

Multimedia Appendix 1 Advanced Secure Cloud Encrypted Platform for Internationally Orchestrated Solutions in Health Care demonstrator for acute stroke care by the Amsterdam University Medical Center. Here, we show how various health care professionals share information about a patient who has experienced a stroke. The information is securely stored in the cloud and becomes available during acute care for the professionals in the emergency call center, ambulance service, and hospital. The fast exchange of information during acute stroke care is essential for making decisions that can have a huge impact on the correct treatment and patient recovery.

Multimedia Appendix 2 Presentation used during the interviews. The videos illustrate the use of the application, similar to the simulations with the participants.

## Abbreviations

ASCLEPIOS: Advanced Secure Cloud Encrypted Platform for Internationally Orchestrated Solutions in Health Care
DSSE: dynamic index-based symmetric searchable encryption
EMR: electronic medical record
G1: group 1
G2: group 2
G3: group 3
G4: group 4
GDPR: General Data Protection Regulation
RQ: research question

## References

[1] Vital M (1999). Stroke Hope Through Research.

[2] Saver J (2006). Time is brain—quantified. Stroke.

[3] Lin MP (2020). Time matters greatly in acute stroke care. Neurol Neurochir Pol.

[4] Oostema JA, Nasiri M, Chassee T, Reeves MJ (2014). The quality of prehospital ischemic stroke care: compliance with guidelines and impact on in-hospital stroke response. J Stroke Cerebrovasc Dis.

[5] Hillestad R, Bigelow J, Bower A, Girosi F, Meili R, Scoville R, Taylor R (2005). Can electronic medical record systems transform health care? Potential health benefits, savings, and costs. Health Aff (Millwood).

[6] Pierantoni G, Kiss T, Terstyanszky G, Dang H, Delgado OS, de OM, Yigzaw K, Belika J, Krefting D, Penzel T (2019). A Secure Cloud-based Platform to Host Healthcare Applications. Proceedings of the 11th International Workshop on Science Gateways, IWSG 2019.

[7] Ren K, Wang C, Wang Q (2012). Security challenges for the public cloud. IEEE Internet Comput.

[8] Yigzaw K, Olabarriaga S, Michalas A, Marco-Ruiz L, Hillen C, Verginadis Y, de Oliveira MT, Krefting D, Penzel T, Bowden J, Bellika J, Chomutare T (2022). Health data security and privacy: challenges and solutions for the future. Roadmap to Successful Digital Health Ecosystems.

[9] General data protection regulation. Intersoft Consulting.

[10] Abbas A, Khan SU (2014). A review on the state-of-the-art privacy-preserving approaches in the e-health clouds. IEEE J Biomed Health Inform.

[11] Munich SA, Tan LA, Nogueira DM, Keigher KM, Chen M, Crowley RW, Conners JJ, Lopes DK (2017). Mobile real-time tracking of acute stroke patients and instant, secure inter-team communication - the join app. Neurointervention.

[12] Nam HS, Heo J, Kim J, Kim YD, Song TJ, Park E, Heo JH (2014). Development of smartphone application that aids stroke screening and identifying nearby acute stroke care hospitals. Yonsei Med J.

[13] Rahmani H, Sundararajan E, Ali ZM, Zin AM (2013). Encryption as a service (EaaS) as a solution for cryptography in cloud. Procedia Technol.

[14] Akinyele J, Pagano M, Green M, Lehmann C, Peterson Z, Rubin A (2011). Securing electronic medical records using attribute-based encryption on mobile devices. Proceedings of the 1st ACM workshop on Security and privacy in smartphones and mobile devices.

[15] Seol K, Kim Y, Lee E, Seo Y, Baik D (2018). Privacy-preserving attribute-based access control model for XML-based electronic health record system. IEEE Access.

[16] Brouwers C, Merten H, Willems M, Habraken DJ, Bloemers FW, Biesheuvel TH, van Galen LS, Nanayakkara PW, Wagner C (2017). Improving care for older patients in the acute setting: a qualitative study with healthcare providers. Neth J Med.

[17] Marinovic S, Craven R, Ma J, Dulay N (2011). Rumpole: a flexible break-glass access control model. Proceedings of the 16th ACM symposium on Access control models and technologies.

[18] Li M, Yu S, Ren K, Lou W (2010). Securing personal health records in cloud computing: patient-centric and fine-grained data access control in multi-owner settings. Security and Privacy in Communication Networks.

[19] Brucker A, Petritsch H, Weber SG (2010). Attribute-based encryption with break-glass. Information Security Theory and Practices. Security and Privacy of Pervasive Systems and Smart Devices.

[20] Yang Y, Zheng X, Guo W, Liu X, Chang V (2019). Privacy-preserving smart IoT-based healthcare big data storage and self-adaptive access control system. Inform Sci.

[21] Advanced secure cloud encrypted platform for internationally orchestrated solutions in healthcare. Asclepios Project.

[22] Michalas A, Bakas A, Dang H, Zalitko A (2019). MicroSCOPE: enabling access control in searchable encryption with the use of attribute-based encryption and SGX. Secure IT Systems.

[23] Bethencourt J, Sahai A, Waters B (2007). Ciphertext-policy attribute-based encryption. IEEE Symposium Security Privacy.

[24] Psarra E, Patiniotakis I, Verginadis Y, Apostolou DG, Mentzas G (2020). Securing access to healthcare data with context-aware policies. Proceedings of the 11th International Conference on Information, Intelligence, Systems and Applications )(IISA.

[25] de Oliveira MT, Bakas A, Frimpong E, Groot AE, Marquering HA, Michalas A, Olabarriaga SD (2020). A break-glass protocol based on ciphertext-policy attribute-based encryption to access medical records in the cloud. Ann Telecommun.

[26] de Oliveira MT, Dang H, A. Reis LH, Marquering HA, D. Olabarriaga S (2021). AC-AC: dynamic revocable access control for acute care teams to access medical records. Smart Health.

[27] Welcome to FHIR. HL7 FHIR.

[28] Chomutare T, Yigzaw K, Olabarriaga S, Makhlysheva A, Oliveira M, Silsand L, Krefting D, Penzel T, Hillen C, Bellika J (2021). Healthcare and data privacy requirements for e-health cloud: a qualitative analysis of clinician perspectives. Proceedings of the IEEE International Conference on E-health Networking, Application & Services, HealthCom 2020.

[29] Reis L, de Oliveira MT, Mattos D, Olabarriaga S (2021). Private data sharing in a secure cloud-based application for acute stroke care. Proceedings of the IEEE 34th International Symposium on Computer-Based Medical Systems (CBMS).

[30] Choi YM (2015). Utilizing end user input in early product development. Procedia Manuf.

[31] Hennink M, Kaiser B (2022). Sample sizes for saturation in qualitative research: a systematic review of empirical tests. Soc Sci Med.

[32] Get insights quickly, with Google forms. Google Forms.

[33] Capture and share insights from your meetings. Otter.

[34] Azevedo V, Carvalho M, Costa F, Mesquita S, Soares J, Teixeira F, Maia A (2017). Interview transcription: conceptual issues, practical guidelines, and challenges. Rev Enf Ref.

[35] Malterud K (2012). Systematic text condensation: a strategy for qualitative analysis. Scand J Public Health.

[36] MAXQDA homepage. MAXQDA.

[37] Murray E, Burns J, May C, Finch T, O'Donnell C, Wallace P, Mair F (2011). Why is it difficult to implement e-health initiatives? A qualitative study. Implement Sci.

[38] Georgiou A, Prgomet M, Lymer S, Hordern A, Ridley L, Westbrook J (2017). The impact of a health IT changeover on medical imaging department work processes and turnaround times. Appl Clin Inform.

[39] Azodo I, Williams R, Sheikh A, Cresswell K (2020). Opportunities and challenges surrounding the use of data from wearable sensor devices in health care: qualitative interview study. J Med Internet Res.

[40] Hasselgren A, Kralevska K, Gligoroski D, Faxvaag A (2021). Medical students' perceptions of a blockchain-based decentralized work history and credentials portfolio: qualitative feasibility study. JMIR Form Res.

[41] Brandt CJ, Clemensen J, Nielsen JB, Søndergaard J (2018). Drivers for successful long-term lifestyle change, the role of e-health: a qualitative interview study. BMJ Open.

[42] Woodward A, Fyfe M, Handuleh J, Patel P, Godman B, Leather A, Finlayson A (2014). Diffusion of e-health innovations in 'post-conflict' settings: a qualitative study on the personal experiences of health workers. Hum Resour Health.

