# Supplementary material for: Perceptions of a Secure Cloud-Based Solution for Data Sharing During Acute Stroke Care: Qualitative Interview Study
Source: JMIR Form Res. 2022 Dec 23;6(12):e40061. doi: 10.2196/40061 (PMC9823575; doi:10.2196/40061)
Supplement: Multimedia Appendix 2 [file formative_v6i12e40061_app2.pptx]

## Slide 1
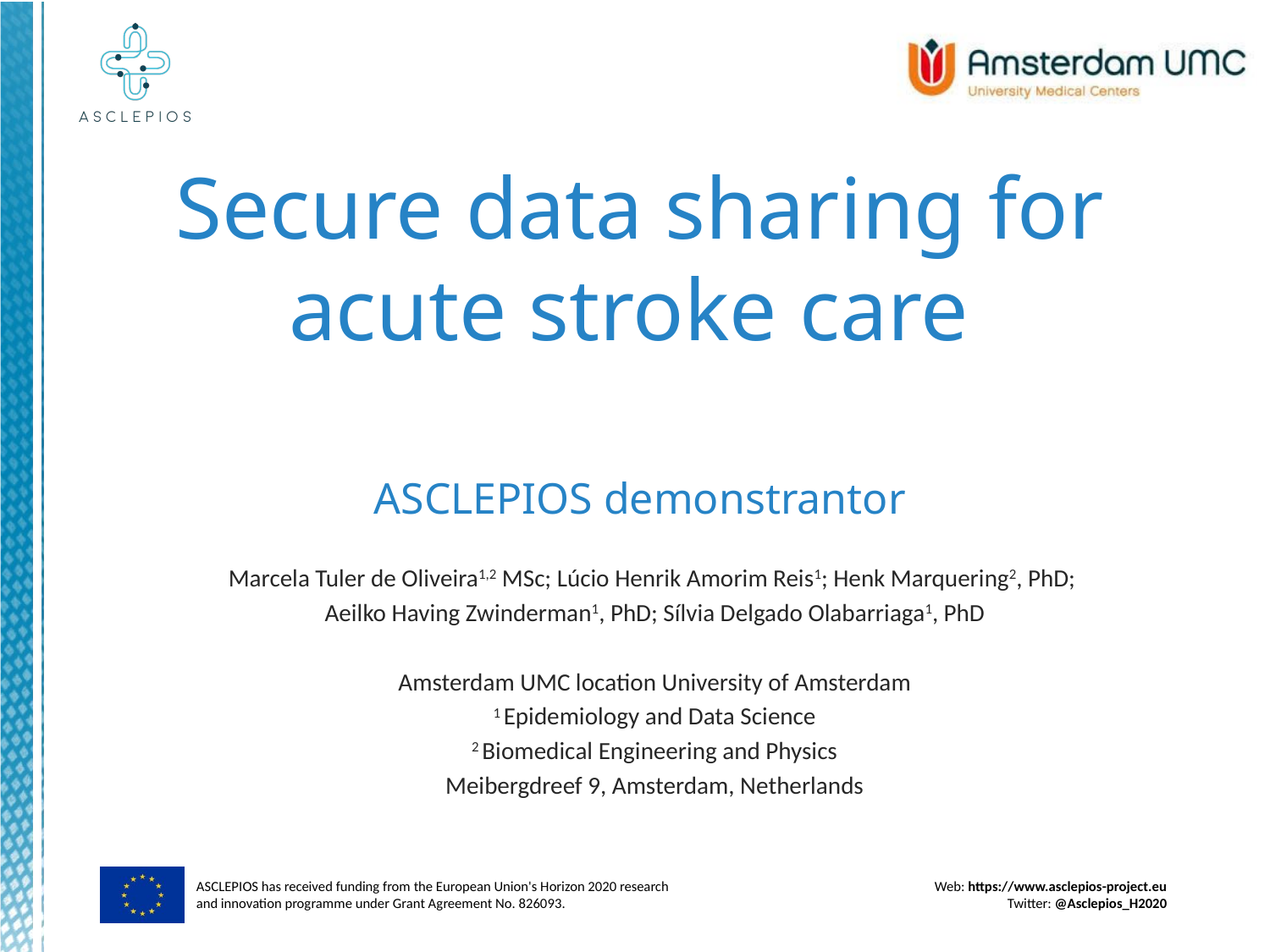

# Secure data sharing for acute stroke care ASCLEPIOS demonstrantor
Marcela Tuler de Oliveira1,2 MSc; Lúcio Henrik Amorim Reis1; Henk Marquering2, PhD;
Aeilko Having Zwinderman1, PhD; Sílvia Delgado Olabarriaga1, PhD
Amsterdam UMC location University of Amsterdam
1 Epidemiology and Data Science
2 Biomedical Engineering and Physics
Meibergdreef 9, Amsterdam, Netherlands

## Slide 2
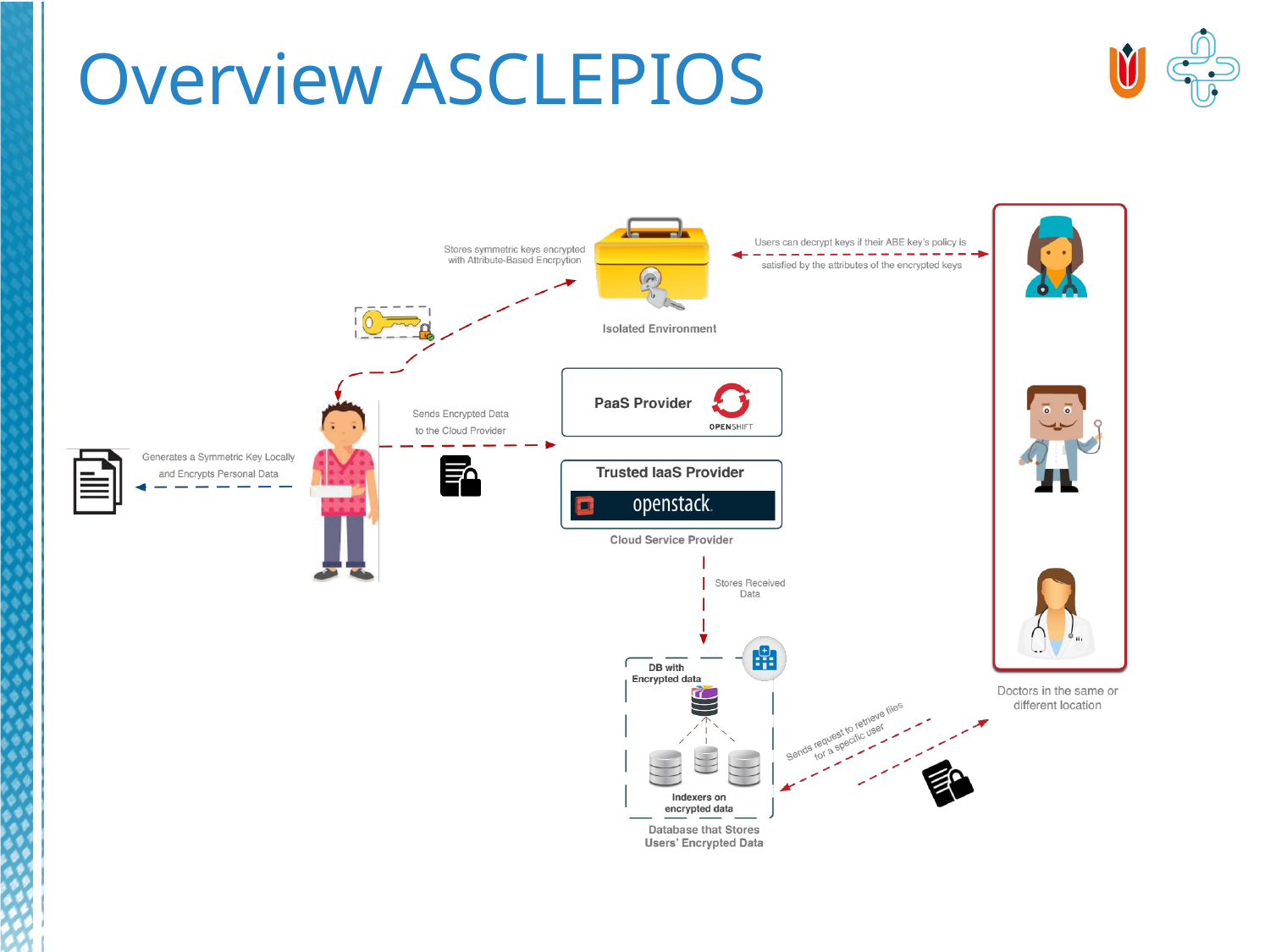

# Overview ASCLEPIOS

## Slide 3
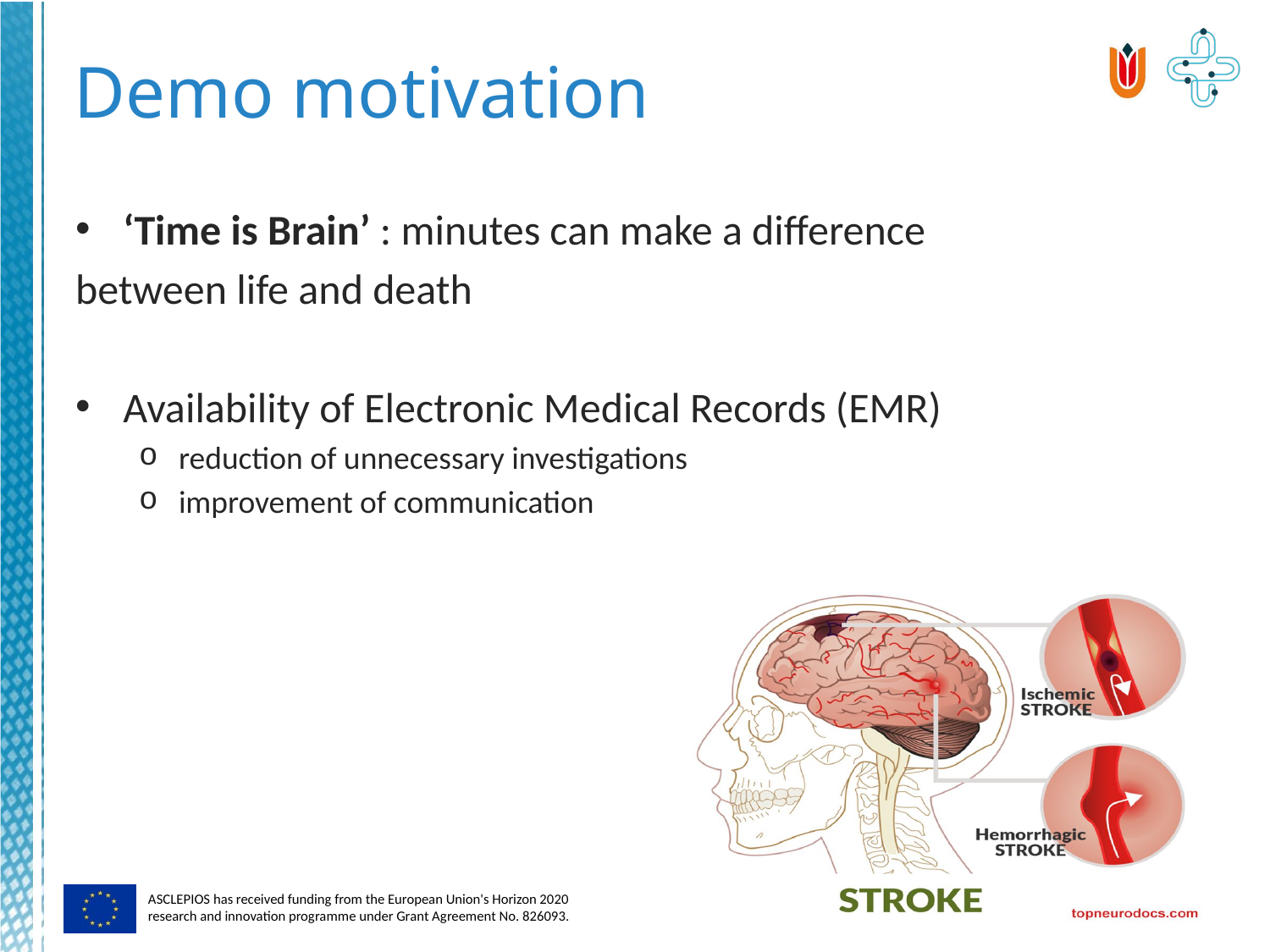

# Demo motivation
‘Time is Brain’ : minutes can make a difference
between life and death
Availability of Electronic Medical Records (EMR)
reduction of unnecessary investigations
improvement of communication
3

## Slide 4
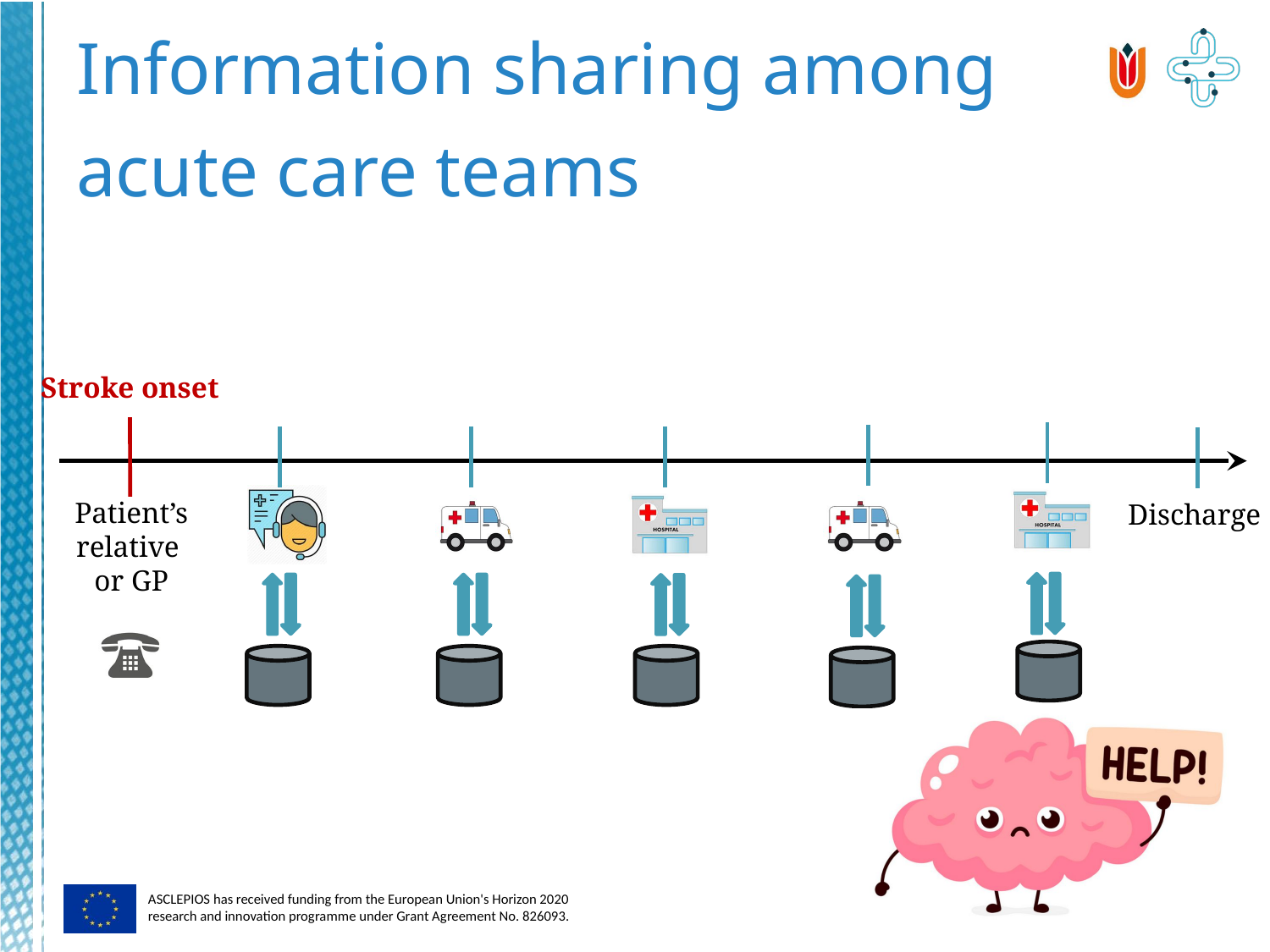

# Information sharing among acute care teams
Stroke onset
Patient’s relative
or GP
Discharge
…
4

## Slide 5
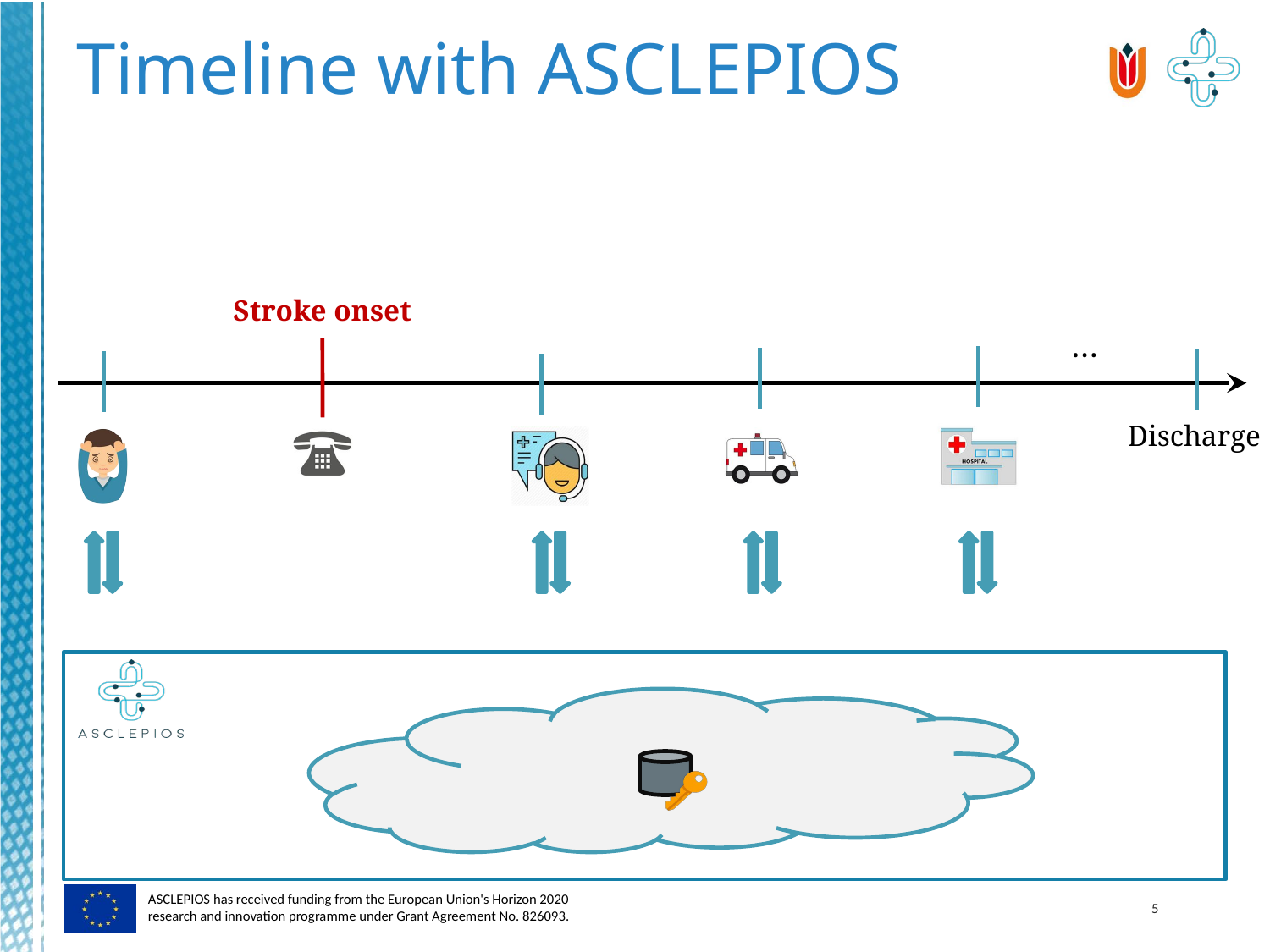

# Timeline with ASCLEPIOS
Stroke onset
…
Discharge
5

## Slide 6
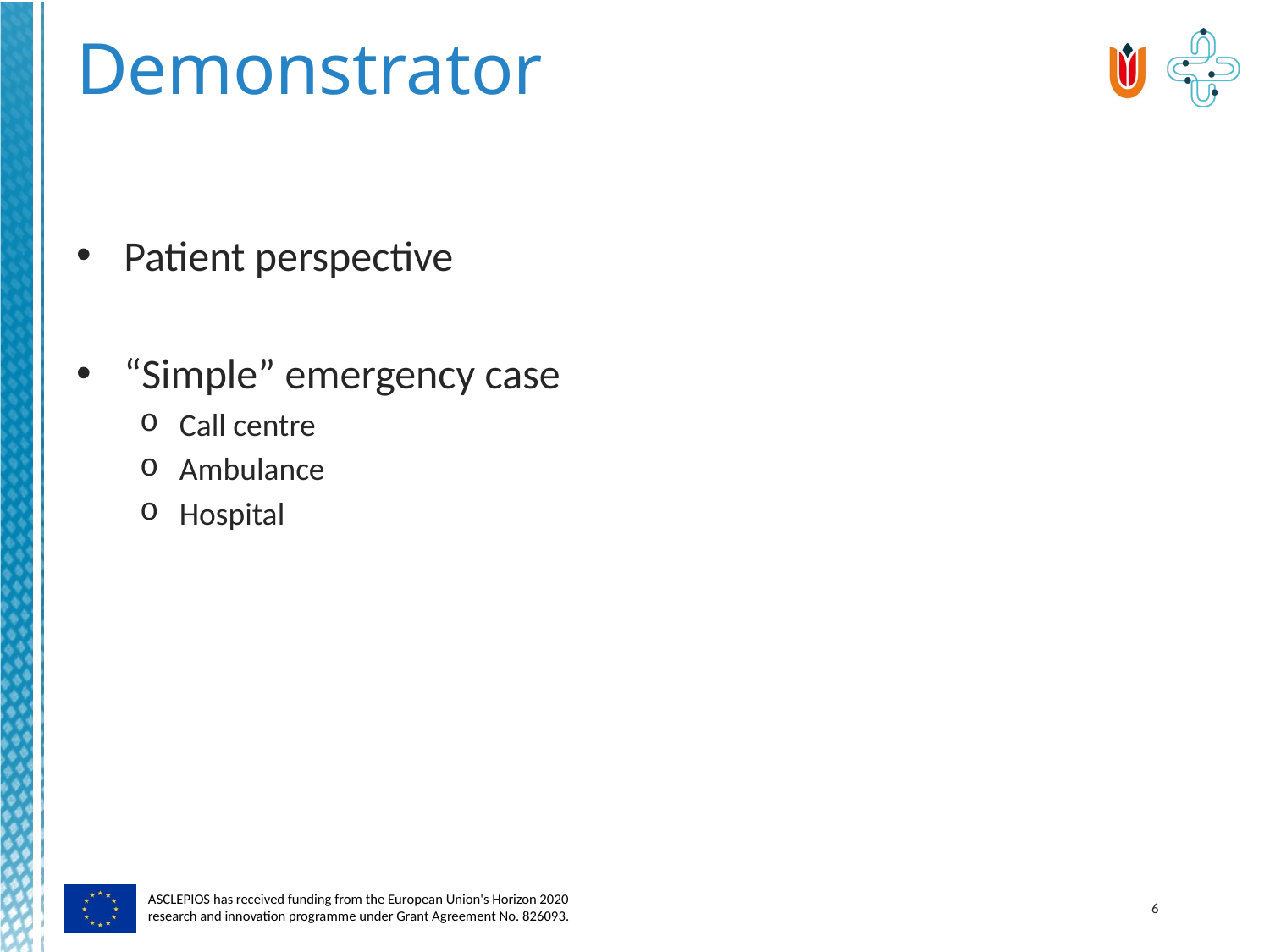

# Demonstrator
Patient perspective
“Simple” emergency case
Call centre
Ambulance
Hospital
6

## Slide 7
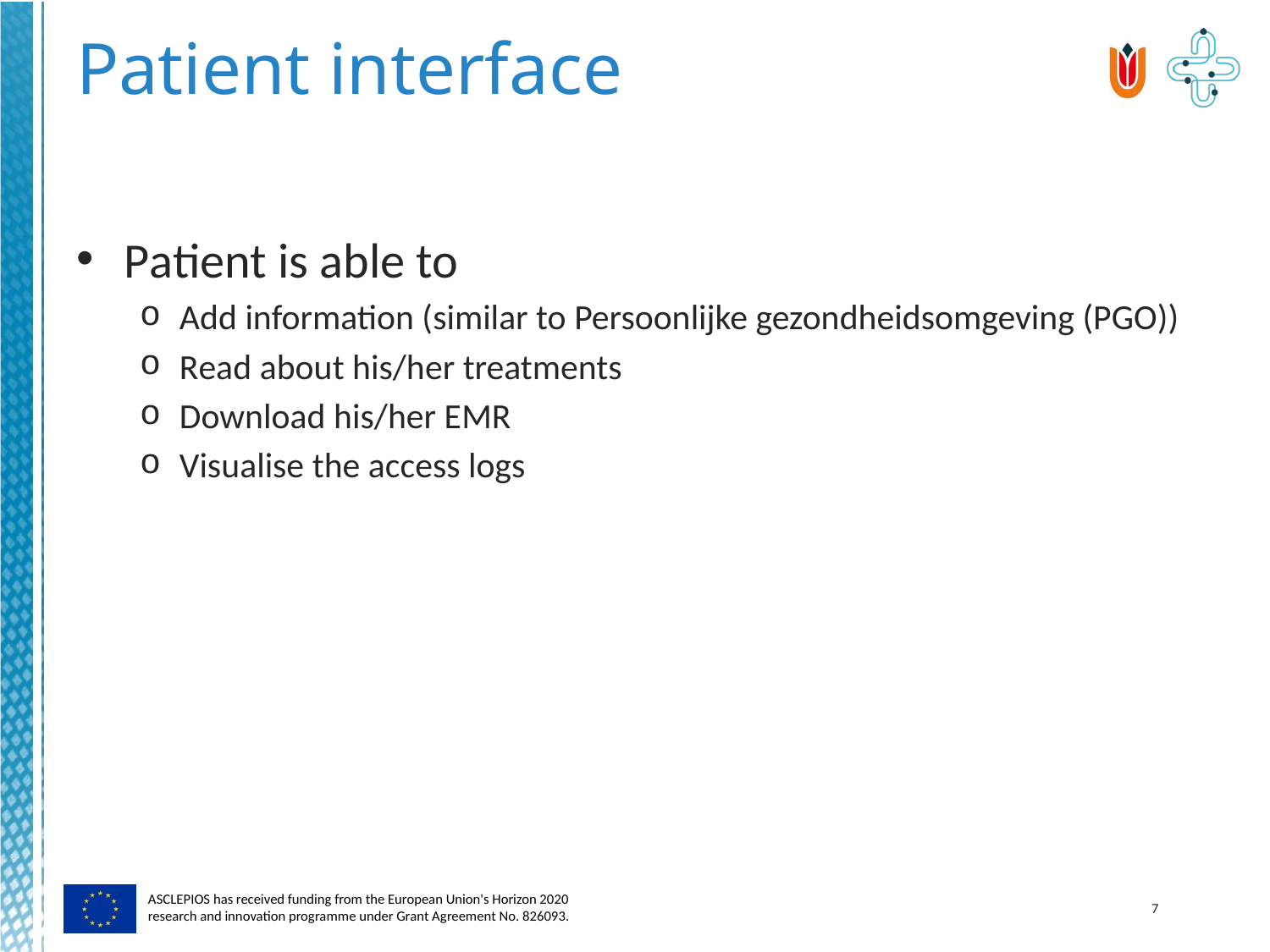

# Patient interface
Patient is able to
Add information (similar to Persoonlijke gezondheidsomgeving (PGO))
Read about his/her treatments
Download his/her EMR
Visualise the access logs
7

## Slide 8
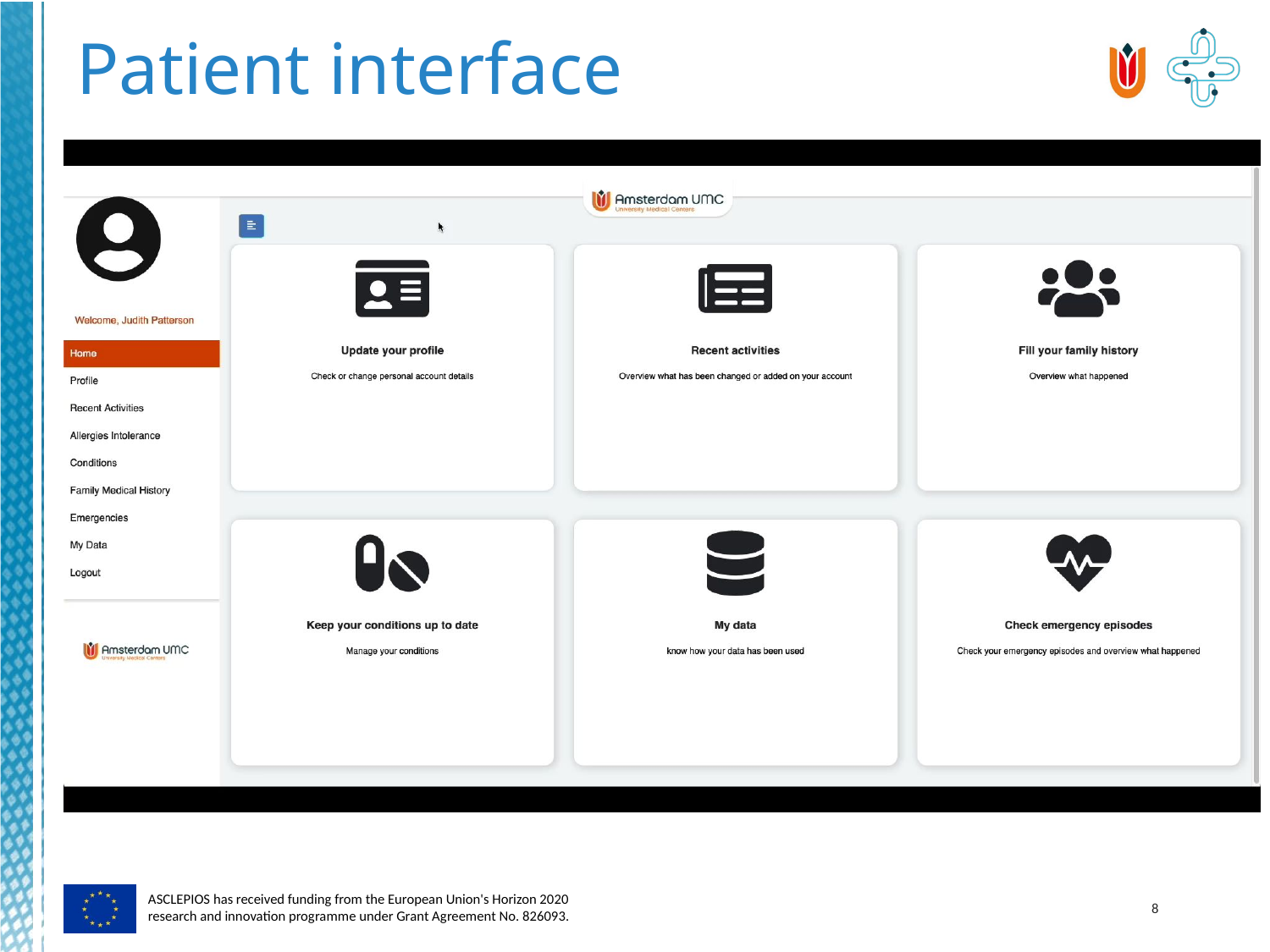

# Patient interface
8

## Slide 9
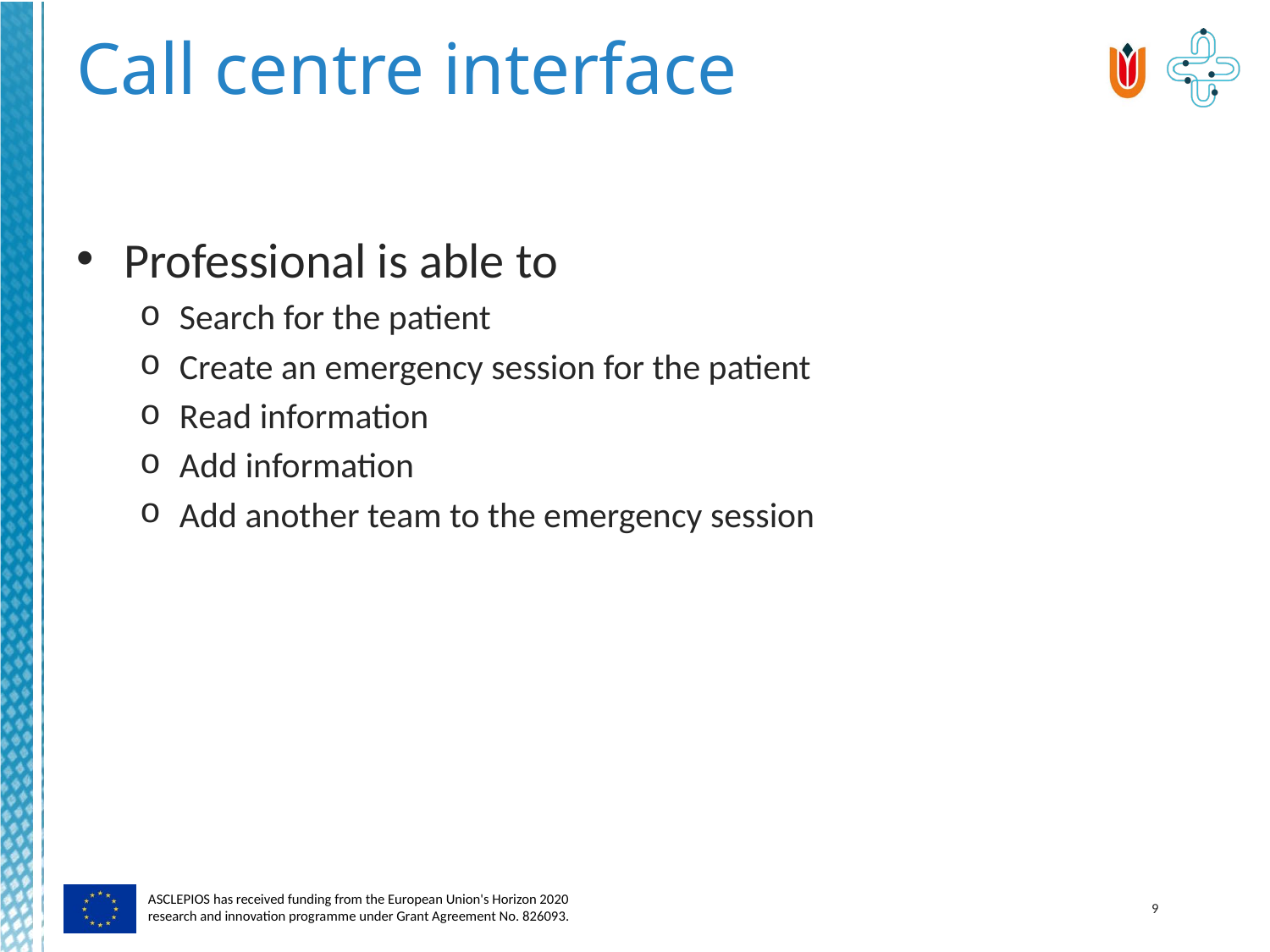

# Call centre interface
Professional is able to
Search for the patient
Create an emergency session for the patient
Read information
Add information
Add another team to the emergency session
9

## Slide 10
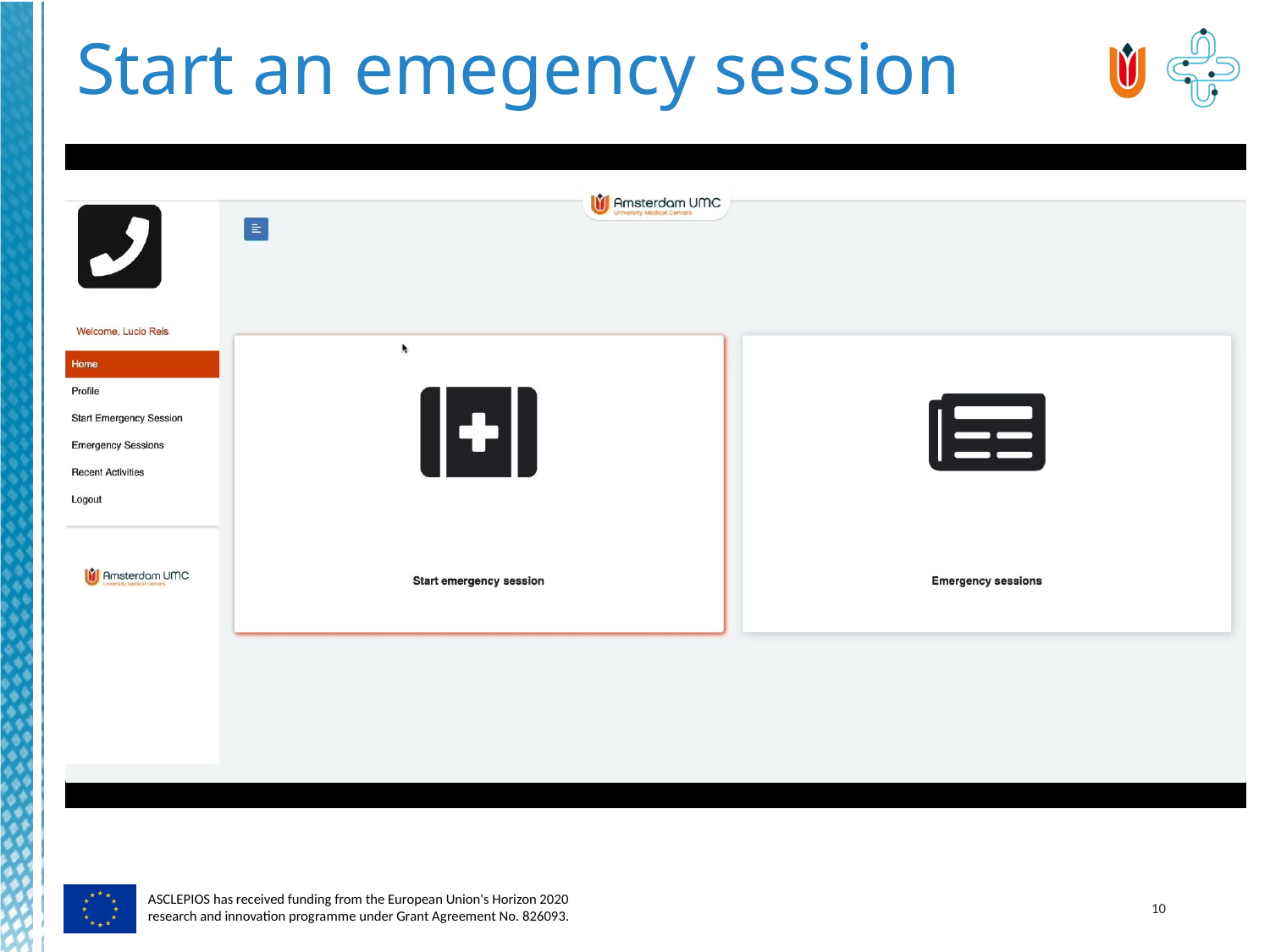

# Start an emegency session
10

## Slide 11
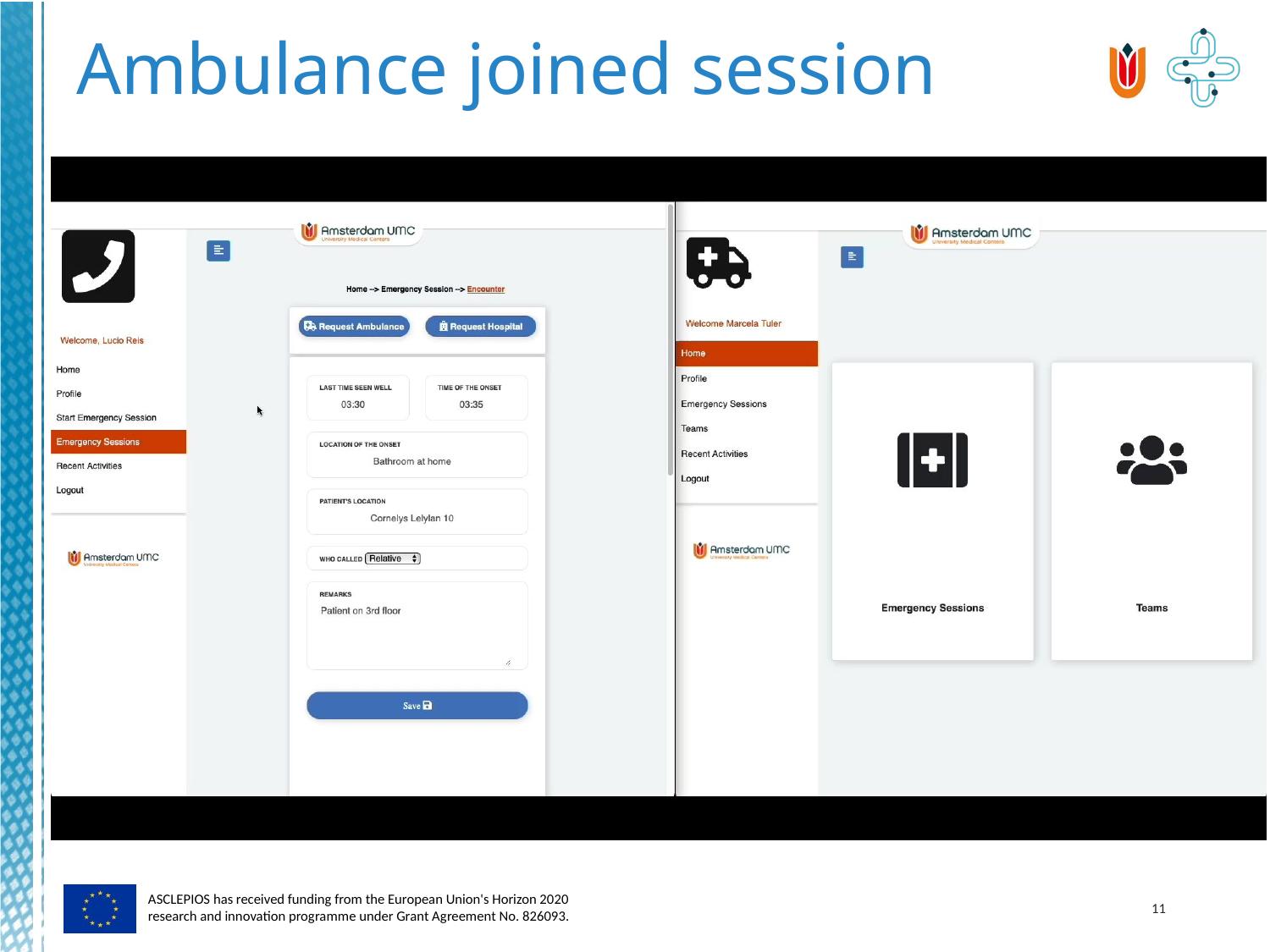

# Ambulance joined session
11

## Slide 12
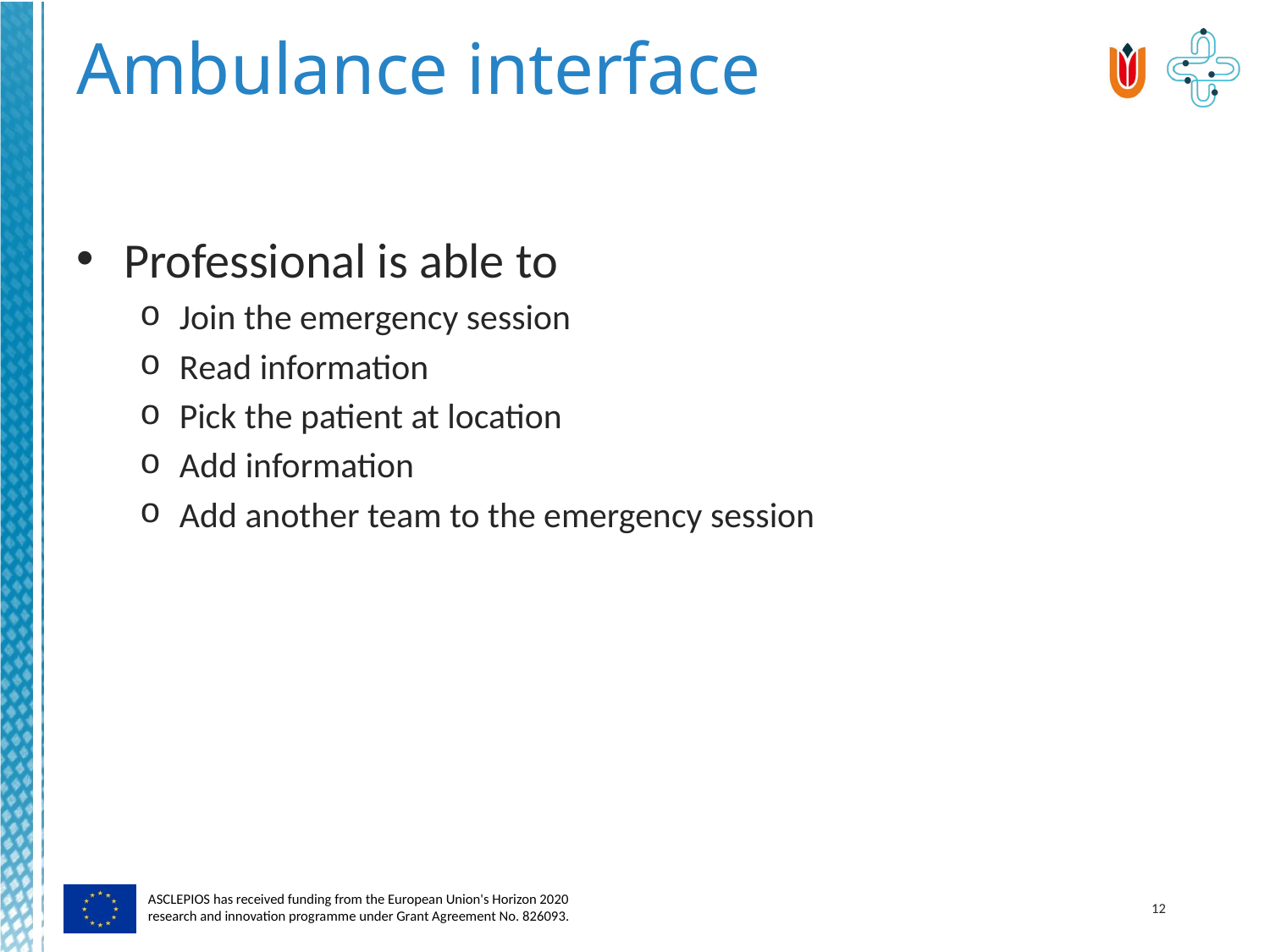

# Ambulance interface
Professional is able to
Join the emergency session
Read information
Pick the patient at location
Add information
Add another team to the emergency session
12

## Slide 13
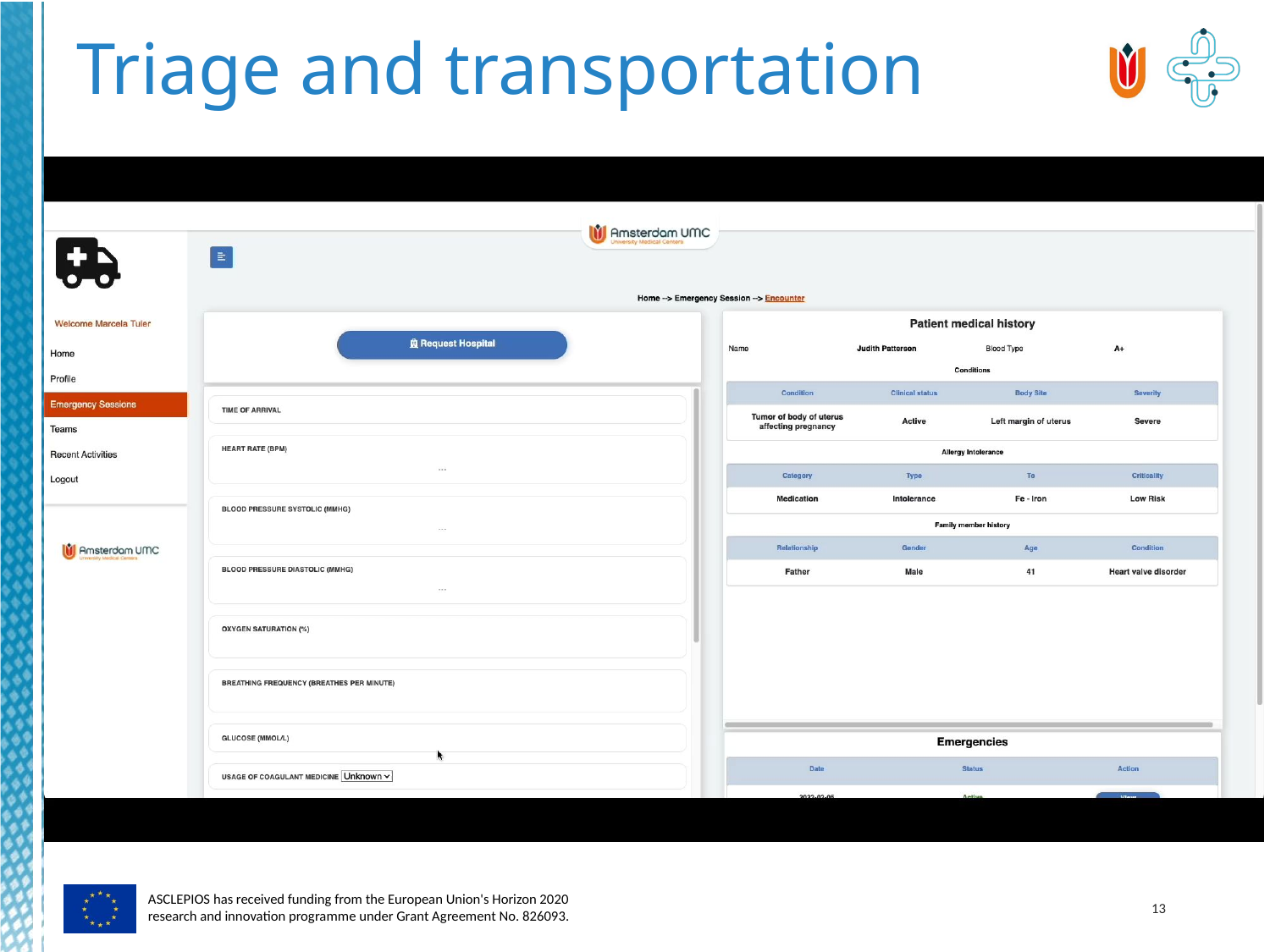

# Triage and transportation
13

## Slide 14
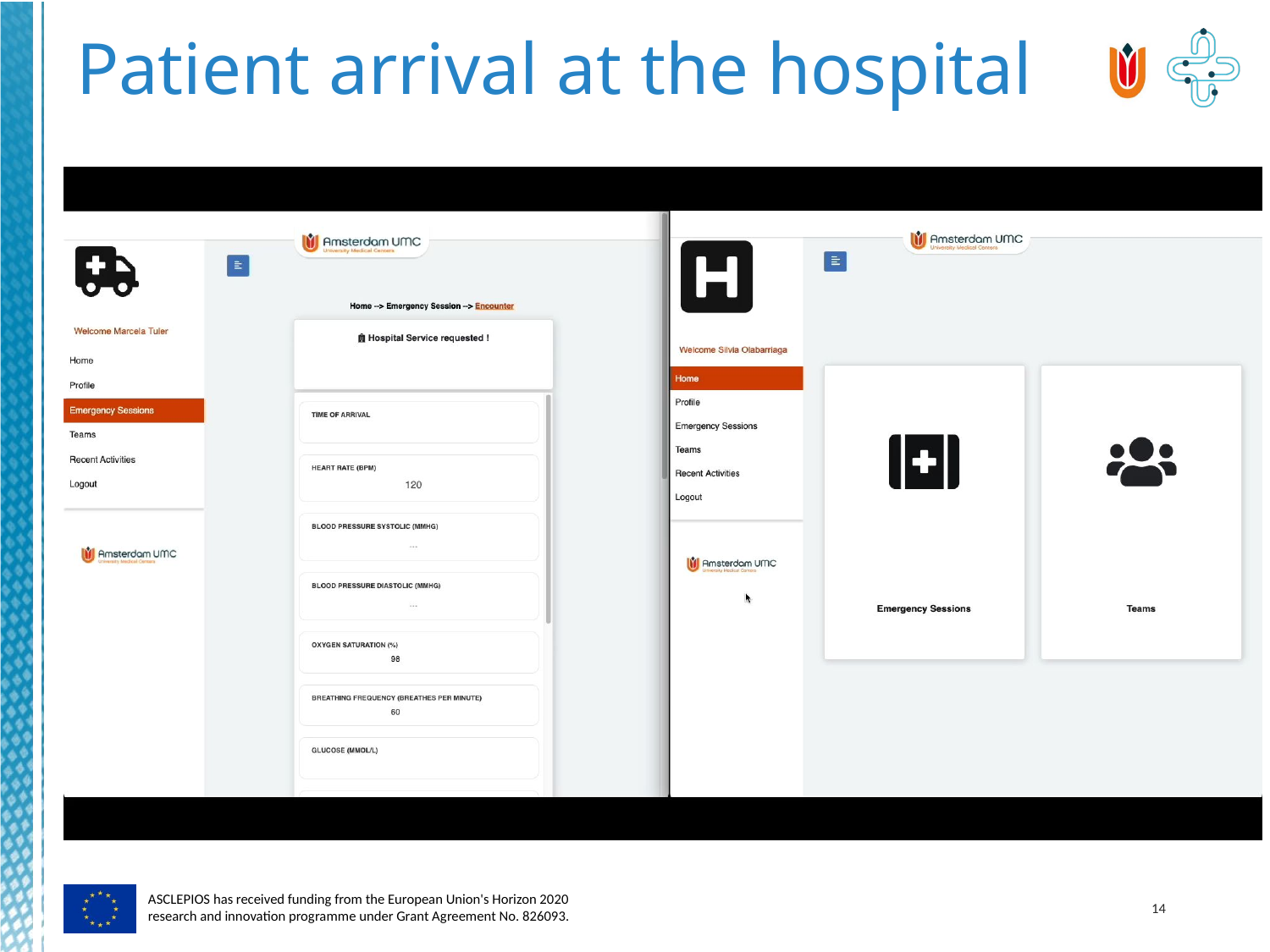

# Patient arrival at the hospital
14

## Slide 15
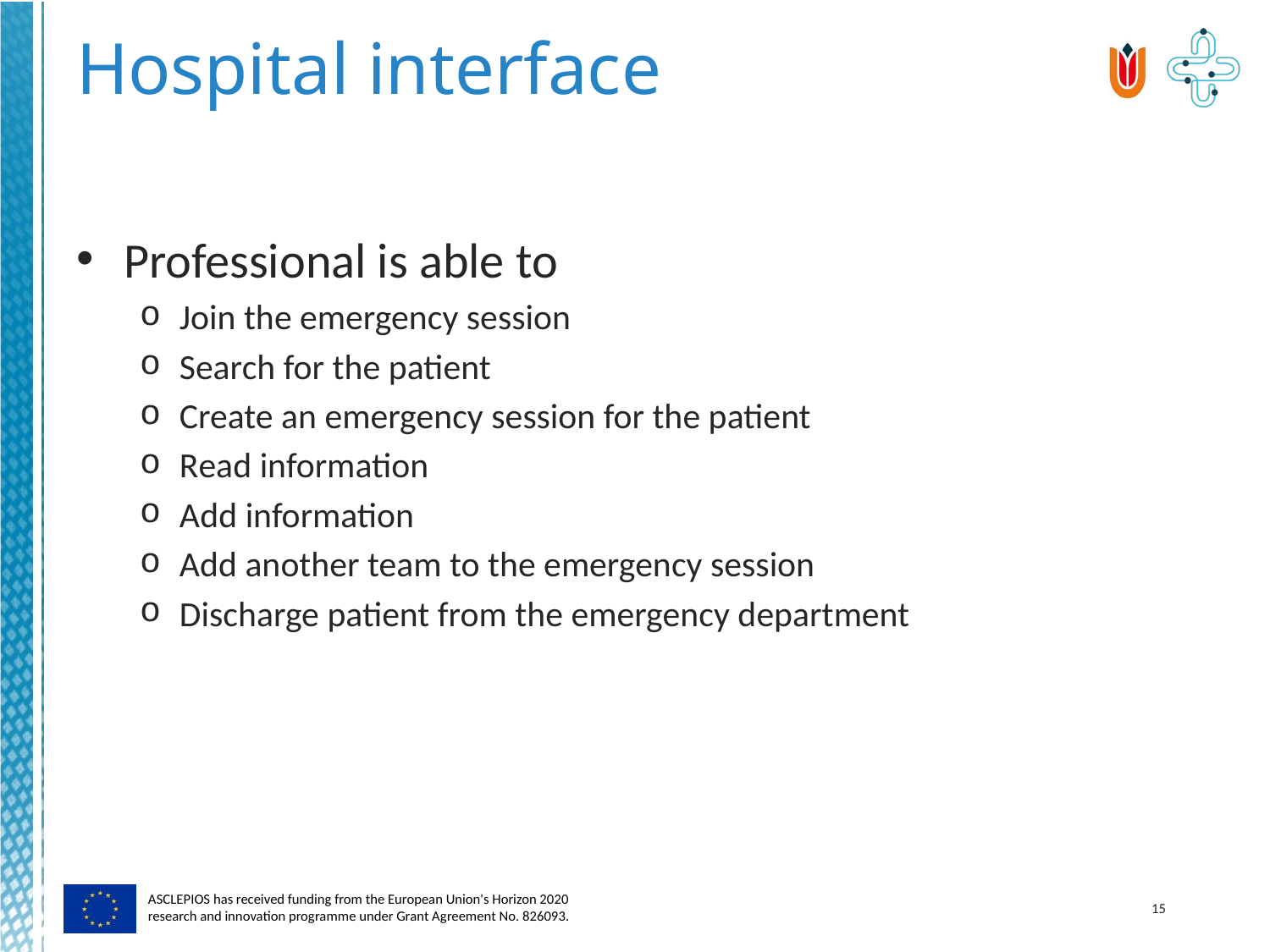

# Hospital interface
Professional is able to
Join the emergency session
Search for the patient
Create an emergency session for the patient
Read information
Add information
Add another team to the emergency session
Discharge patient from the emergency department
15

## Slide 16
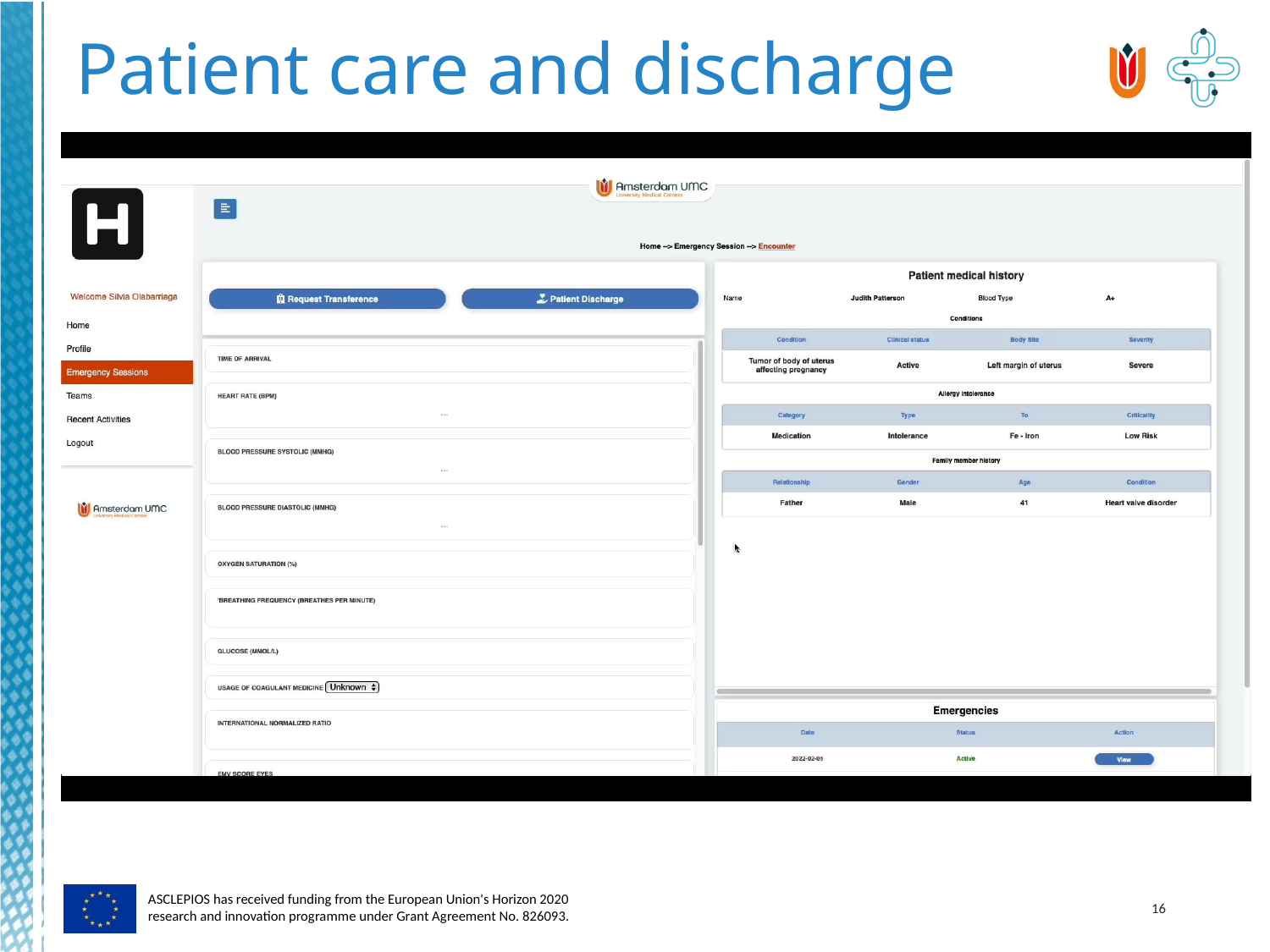

# Patient care and discharge
16

## Slide 17
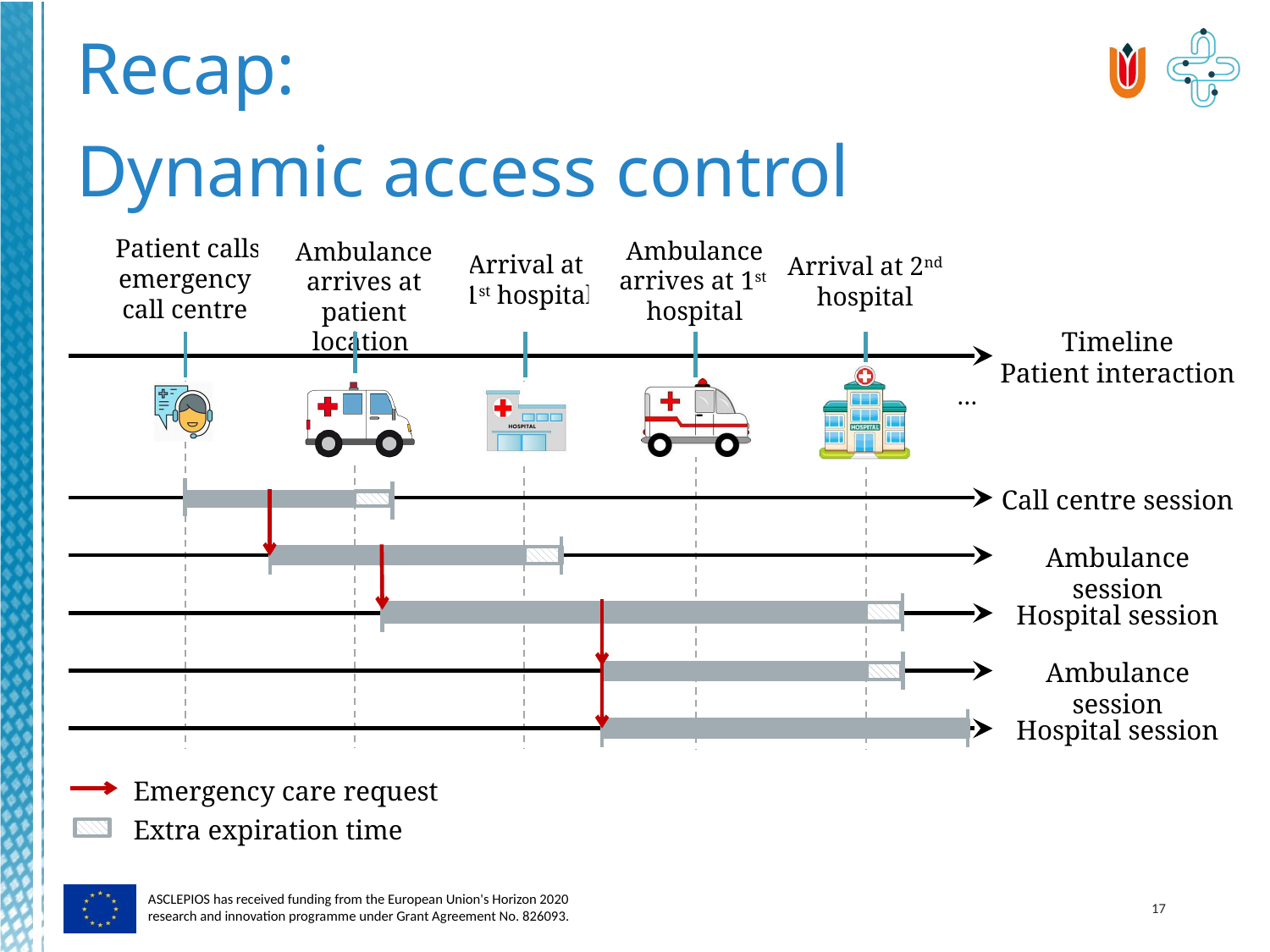

# Recap: Dynamic access control
Patient calls emergency
call centre
Ambulance arrives at 1st
hospital
Ambulance arrives at patient location
Arrival at
 1st hospital
Arrival at 2nd hospital
Timeline
Patient interaction
…
Call centre session
Ambulance session
Hospital session
Ambulance session
Hospital session
Emergency care request
Extra expiration time
17

## Slide 18
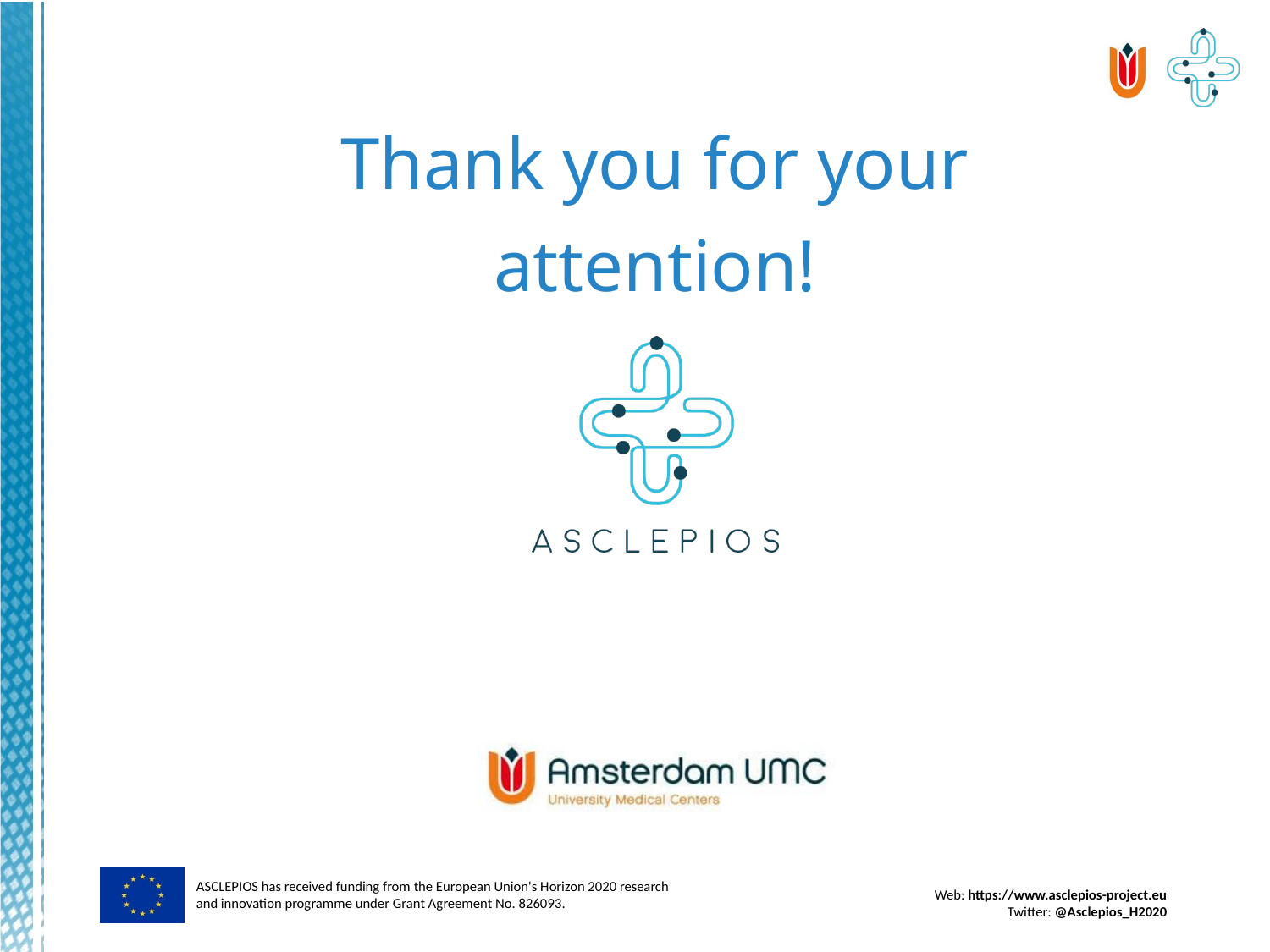

# Thank you for your attention!
